# Improving the management procedures in farms infected with the Porcine Reproductive and Respiratory Syndrome virus using PDP models

**DOI:** 10.1038/s41598-019-46339-w

**Published:** 2019-07-10

**Authors:** Ma Àngels Colomer, Antoni Margalida, Lorenzo Fraile

**Affiliations:** 1 Department of Mathematics ETSEA, University of Lleida, 25198 Lleida, Spain; 2 Department of Animal Science, ETSEA, University of Lleida, 25198 Lleida, Spain; 3 Institute for Game and Wildlife Research, IREC (CSIC-UCLM-JCCM), 13005 Ciudad Real, Spain; 4 Agrotecnio, University of Lleida, 25198 Lleida, Spain

**Keywords:** Respiratory tract diseases, Computational models

## Abstract

Pig meat production need to be built up in the future due to the increase of the human population worldwide. To address this challenge, there is plenty of room for improvement in terms of pig production efficiency that could be severely hampered by the presence of diseases. In this sense, Porcine Reproductive and Respiratory Syndrome Virus (PRRSV) is one of the most costly disease present in industrial pork production in Europe and North America. We have developed a model to analyze the effect of different management procedures to control this important virus in different epidemiological scenarios. Our results clearly suggest that no cross-fostering during lactation and the maintaining of litter integrity significantly decrease the number of sick and dead animals during the rearing period compared to scenarios where cross-fostering and no litter integrity are practiced. These results highlight the relevance of different management strategies to control PRRSV and quantify the effect of limiting cross-fostering and avoiding mixing animals from different litters in PRRSV positive farms to optimize animal production. Our findings will allow pig farmers to apply these management procedures to control this disease under field conditions in a very cost-effective way.

## Introduction

The United Nations Food and Agriculture Organization (FAO) projects that food production must increase by 70% to feed the world’s population by the year 2050^1^. Pig meat is one of the most important sources of animal protein for humans and a sustained increased in pig production will be necessary to cope with the challenge of providing enough food worldwide. This challenge can be tackled either by increasing the number of animals and/or by improving the efficiency of the pork production sector. Diseases have multiple effects on the performance of affected animals since nutrients are necessary to fight against disease. Consequently, sick animals are less efficient to transform food into edible products. Thus, it is necessary to use techniques such as gross margin analysis or partial budgeting to evaluate the economic consequences of diseases on animal performance in order to select the most suitable measures based on the best cost/benefit ratios^2^.

PRRSV virus causes considerable economic losses and it is probably the most costly disease affecting industrial pork production in Europe and North America^3^. Thus, this disease annually causes losses of $664 million to the US pork industry^4^. The most common clinical symptoms, following PRRSV infections, include reproductive failure, respiratory disease in young pigs and reduced growth^5,6^. Preventive medicine programs to control PRRSV are commonly based on performing gilt acclimation protocol, a PRRSV vaccination program for gilts and sows, and different management protocols to control the prevalence of pathogens in suckling pigs. On the other hand, eradication methods can be developed using different techniques at farm or even at regional level^7^. The first step is to decide whether the best approach is to control or eradicate the virus from a PRRSV positive farm. In this sense, more information is needed to have update information about the economic cost of PRRSV in each particular pig production system and to choose the best option in a case by case situation.

One way to control disease and enhance performance efficiency is to improve animal health by implementing appropriate management methods focused on limiting the transmission of infectious agents within the population. In the case of PRRSV, the rationale of this approach is the need to apply suitable management procedures such as a decrease in the mixing of animals during the rearing cycle to decrease the spread of the virus and, consequently, diminish its negative effects on pig productivity. However, the large-scale practical application of management procedures is highly complex and it is difficult to obtain comparable information regarding the implications that such procedures have for disease control and their economic consequences. Thus, a modeling framework in which to test the different management procedures used to control PRRSV would be of great value. Mathematical models can provide new knowledge about the epidemiology of infectious diseases and criteria to design more efficient control strategies^8^. These models are tools for understanding key points in epidemiology such as disease transmission and dynamics since the implications of the spread of pathogens and the outcome of “what if” scenarios can be used to predict the effects of future interventions^9–11^. To date, this type of modeling has usually been carried out using stochastic models for both PRRSV and other pig diseases^12–14^. The Population dynamic P system (PDP) models are an alternative to traditional modeling (bioinspired by cell functioning) that have never been applied in swine epidemiology until now. Briefly, cells are able to carry out multiple processes simultaneously in a synchronized fashion, which makes them a suitable dummy when it is necessary to model complex problems. Thus, new emerging generations of computational models such as PDP models are useful tools to study complex problems with a huge number of interactions in a more affordable way^15^. Here, a stochastic model is developed based on PDP models that mimic the intra-herd dynamics of a standard pig production system in order to disentangle the consequences of pig-farm management measures on the control of PRRSV virus under several different epidemiologic scenarios.

## Results

### Uncertainty and sensitivity analyses

As a variable response in the Box-Bhenken design, the percentage of sick animals at the end of lactation, nursery, and fattening period were used. Therefore, three response surfaces were obtained (Supporting Information). The percentage of infected sows, *R*_0_ and lethality are the parameters that have a significant effect on the percentage of sick animals at the edge of the nursery and fattening period. However, the probability of transmission of the disease during the lactation phase slightly affects during this production phase. Using the response surfaces obtained, the sensitivity was studied in all three phases of the pig breeding process (Supporting Information).

The increase of infected piglets is very small (from −3‰ to 3‰) during the lactation phase in the range of values used for infected sows. However, in the nursery (from 1.5 to 5.9%) and fattening phase (from 0.5 to 5.77%), this increase is bigger using low versus high level of infected sows. During the rearing period (nursery and fattening), an increase of one point (1%) in the percentage of infected sows implied an increase between 3 and 6% of infected pigs. In the latter two cases, the value decreases as the percentage of infected sows increases (Supporting Information).

In the case of the *R*_0_ parameter, an increase of one unit implies similar variations in the percentage of sick animals during the nursery (from 2.1 to 5.8%) and fattening phase (from 2.6 to 6.2%) in the range of studied values. However, in the lactation phase, this increase is practically nil (from 0.02 to −0.94%).

In the case of variations in the lethality value, the increase in sick animals does not practically vary during the nursery (from −0.9 to 0.1%) and fattening period (from −0.7 to 0.1%) in the range of studied values. In the lactation phase, it increases as the lethality increases, but the variations are very small (from −1.2 to 1.3%).

### Porcine Reproductive and Respiratory Syndrome (PRRSV) model

Cross-fostering (CF), the proportion of infected sows during lactation and the management carried out during nursery and fattening affect the proportion of infected animals found at the end of nursery and fattening, as well as the total number of animals infected by the disease during the whole rearing period (including dead animals) (Tables 1, 2).

**Table 1:** Morbidity and mortality at the end of nursery and fattening period taking into account lactation (cross-fostering) and management procedures during the rearing period.

Proportion of infected sows		Management	No CF (%)	CF1 (%)	CF3 (%)
0.01	Morbidity at the end of the transition phase	MP	7.6	7.5	10.2
		RR	8.4	7.9	10.6
	Morbidity at the end of the fattening phase	MP	7.4	7.0	9.6
		RR	8.6	7.6	10.3
	Morbidity and mortality during transition and fattening phase	MP	19.5	18.2	20.9
		RR	20.8	18.9	21.7
0.025	Morbidity at the end of the transition phase	MP	19.2	25.9	28.0
		RR	19.6	25.9	26.4
	Morbidity at the end of the fattening phase	MP	18.1	24.2	26.0
		RR	19.0	25.0	26.8
	Morbidity and mortality during transition and fattening phase	MP	30.1	35.8	37.7
		RR	31.3	36.9	38.9
0.05	Morbidity at the end of the transition phase	MP	34.3	40.8	46.4
		RR	34.2	40.7	44.8
	Morbidity at the end of the fattening phase	MP	32.0	37.7	42.2
		RR	32.6	38.6	41.4
	Morbidity and mortality during transition and fattening phase	MP	43.6	49.8	54.6
		RR	44.5	51.0	54.5
0.1	Morbidity at the end of the transition phase	MP	42.7	63.1	64.1
		RR	42.7	59.6	60.9
	Morbidity at the end of the fattening phase	MP	39.9	55.6	56.1
		RR	40.5	53.2	53.8
	Morbidity and mortality during transition and fattening phase	MP	54.3	69.8	70.6
		RR	54.7	68.6	69.2

Morbidity is calculated as the percentage of infected animals versus the whole population during one period and mortality is calculated as the percentage of dead animals versus the whole population during one period.

CF means cross-fostering. CF1 and CF3 mean that cross-fostering is carried out one or three times during the lactation period. MP means orderly movements consisting of filling the pens during transition according to the sow’s origin and during fattening according to nursery distribution whereas RR means movements consisting of filling the nursery and fattening pens at random.

**Table 2:** ANOVA analysis results on the effect of management during the lactation (cross-fostering), transition and fattening period on morbidity and mortality at the end of the transition and fattening period.

Proportion infected sows		CF		Management		CF*management	
		F	P-value	F	P- value	F	P-value
0.01	Morbidity at the end of the transition phase	126.21	<**0.001**	17.21	<**0.001**	0.85	0.43
	Morbidity at the end of the fattening phase	74.94	<**0.001**	32.71	<**0.001**	1.21	0.30
	Morbidity and mortality during transition and fattening phase	118.48	<**0.001**	63.59	<**0.001**	2.31	0.11
0.025	Morbidity at the end of the transition phase	817.77	<**0.001**	2.37	0.1276	0.44	0.64
	Morbidity at the end of the fattening phase	510.1	<**0.001**	17.12	<**0.001**	0.012	0.99
	Morbidity and mortality during transition and fattening phase	708.14	<**0.001**	42.01	<**0.001**	0.04	0.96
0.05	Morbidity at the end of the transition phase	693.2	<**0.001**	3.33	0.072	2.99	0.56
	Morbidity at the end of the fattening phase	482.49	<**0.001**	1.7	0.1969	3.09	**0.051**
	Morbidity and mortality during transition and fattening phase	380.59	<**0.001**	11.16	**0.001**	2.82	0.066
0.1	Morbidity at the end of the transition phase	3040.65	<<**0.001**	45.56	<**0.001**	22.37	<**0.001**
	Morbidity at the end of the fattening phase	1393.49	<**0.001**	9.21	**0.0033**	14.50	<**0.001**
	Morbidity and mortality during transition and fattening phase	2954.50	<**0.001**	5.53	**0.0214**	10.01	**0.00014**

Morbidity is calculated as the percentage of infected animals versus the whole population during one period and mortality is calculated as the percentage of dead animals versus the whole population during one period. In bold type appear statistically significant results.

CF means cross-fostering. Management refers to orderly or random movements during transition and fattening. F means the statistical contrast value.

Performing CF significantly increases both, the proportion of animals that are infected at the end of nursery and fattening phase and the total number of animals affected by the disease during the rearing period. Moreover, these morbidity parameters significantly increase with the proportion of positive sows (Tables 1, 2). However, the management carried out during nursery and fattening does seem to have differing effects depending on the number of sows which can transmit the disease. Thus, in the case of low disease prevalence in sows (1%), the three parameters previously described are significantly reduced if the animals are managed by litters (litter integrity). On the other hand, with a prevalence of 2.5% of infected sows, a significantly lower percentage is only observed in the case of infected pigs at the end of fattening and during the whole cycle if the litter effect is maintained throughout the rearing period.

**Figure 1:**
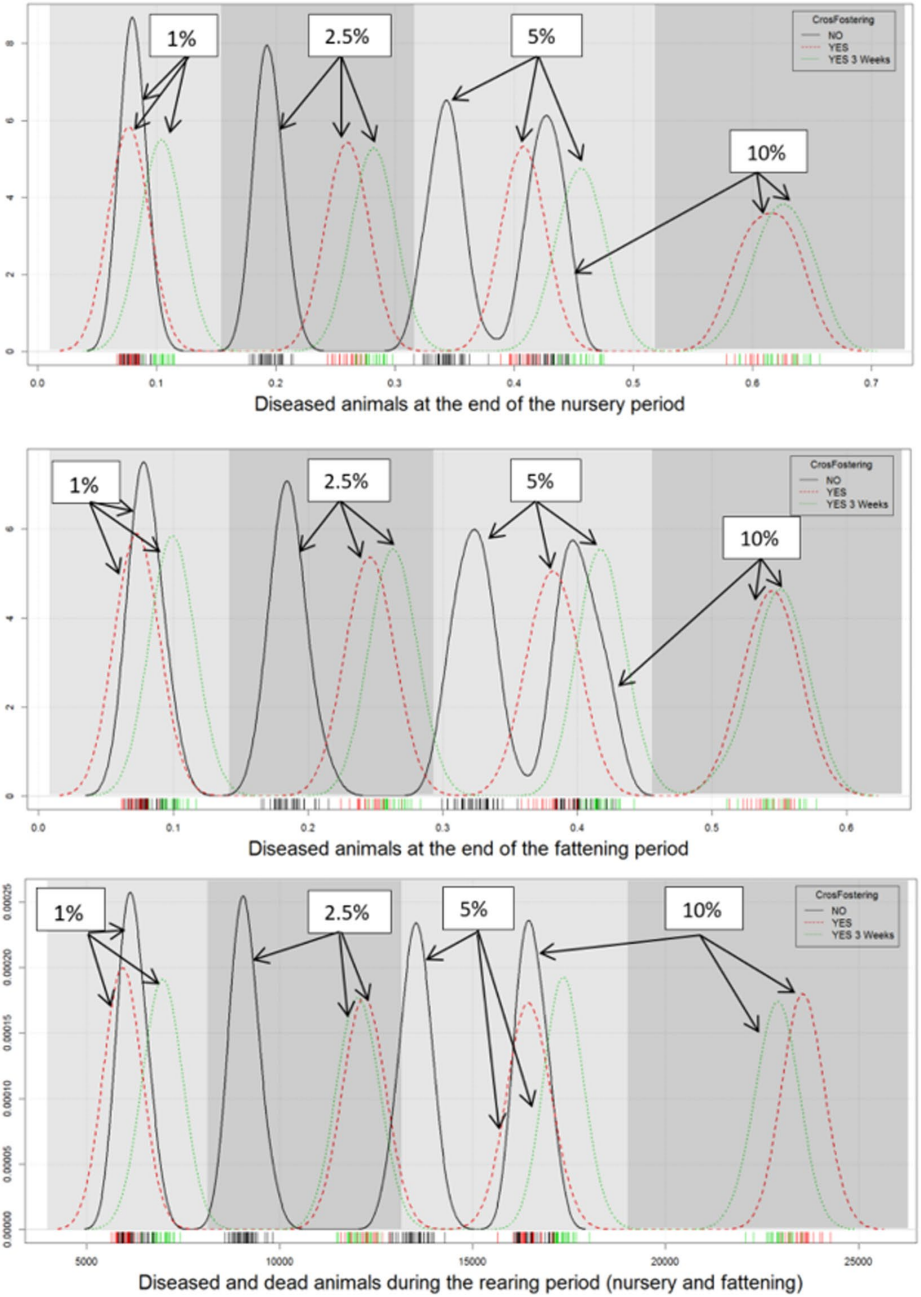
Density function of the infected animals at the end of the nursery and fattening period. Moreover, it is also represented the infected and dead animals during the whole rearing period.

When the proportion of infected sows reaches 5%, the management applied during nursery and fattening does not significantly affect the number of pigs that are infected at the end of these phases. Moreover, if the proportion of infected sows is less than or equal to 5%, the management performed always significantly affects the total number of infected animals during lactation, which increases in the case of random management during nursery and fattening. However, when the proportion of infected sows rises to 10%, the results are reversed, that is, with randomization during nursery and fattening, fewer animals are infected when they reach the nursery and fattening phases because the disease has already spread to the population during the lactation period and the first transition phase (Fig. 1).

In general terms, the mortality is significantly lower in the nursery and fattening phase if no cross-fostering is carried out versus carrying out this management procedure during the lactation phase regardless the percentage of PRRSV infected sows. However, the mortality is only significantly lower during the fattening phase if litter integrity is applied versus carrying out a random process to allocate pigs during the rearing period Finally, it was no observed any significant interaction between cross-fostering and litter integrity management for mortality parameters during the rearing period (Tables 3, 4).

**Table 3:** Mortality (%) in nursery and fattening taking into account cross-fostering and management procedures. Mortality is calculated as the percentage of dead animals versus the whole population during one period.

Proportion of infected sows		Management	No CF	CF1	CF3
0.01	Mortality at the end of the transition phase	MP	0.04	0.10	0.14
		RR	0.05	0.09	0.14
	Mortality at the end of the fattening phase	MP	0.010	0.006	0.007
		RR	0.02	0.008	0.15
0.025	Mortality at the end of the transition phase	MP	0.15	0.41	0.48
		RR	0.16	0.43	0.47
	Mortality at the end of the fattening phase	MP	0.01	0.02	0.02
		RR	0.02	0.04	0.03
0.05	Mortality at the end of the transition phase	MP	0.36	0.74	0.99
		RR	0.36	0.74	0.97
	Mortality at the end of the fattening phase	MP	0.01	0.03	0.02
		RR	0.03	0.04	0.04
0.1	Mortality at the end of the transition phase	MP	0.40	1.8	1.8
		RR	0.40	1.8	1.9
	Mortality at the end of the fattening phase	MP	0.02	0.03	0.04
		RR	0.03	0.05	0.06

CF means cross-fostering. Management refers to orderly or random movements during transition and fattening. CF1 and CF3 means that cross-fostering is carried out one or three times during the lactation period. MP means orderly movements consisting of filling the pens during transition according to the sow’s origin and during fattening according to nursery distribution whereas RR means movements consisting of filling the nursery and fattening pens at random.

**Table 4:** ANOVA analysis results on the effect of management during the lactation (cross-fostering), transition and fattening period on mortality at the end of the transition and fattening period. Mortality is calculated as the percentage of dead animals versus the whole population during one period. In bold type appear statistically significant results.

Proportion infected sows		CF		Management		CF*management	
		F	P-value	F	P- value	F	P-value
0.01	Mortality at the end of the transition phase	152.79	<**0.001**	0.20	0.65	0.78	0.46
	Mortality at the end of the fattening phase	6.97	<**0.001**	22.08	<**0.001**	2.28	0.11
0.025	Mortality at the end of the transition phase	376.08	<**0.001**	0.32	0.58	0.74	0.48
	Mortality at the end of the fattening phase	7.50	<**0.001**	31.53	<**0.001**	1.48	0.23
0.05	Mortality at the end of the transition phase	290.43	<**0.001**	0.08	0.77	0.08	0.93
	Mortality at the end of the fattening phase	4.78	**0.015**	36.52	<**0.001**	0.97	0.39
0.1	Mortality at the end of the transition phase	1516.16	<**0.001**	0.37	0.54	0.35	0.70
	Mortality at the end of the fattening phase	22.41	<**0.001**	24.21	<**0.001**	0.25	0.78

CF means cross-fostering. Management refers to orderly or random movements during transition and fattening. F means the statistical contrast value.

### Validation of the model with farm data

We obtained empirical data from two farms to validate the model. The included farms were PRRSV positive without observing overt PRRSV clinical outbreaks in the last twelve months. Thus, samples from adult breeding animals, weaning-age pigs and growing pigs were negative by PCR (shedding status) during the study period. On the other hand, most of the tested sera (>80%) in both phases were positive for PRRSV antibody in adult breeding animals, weaning-age pigs and growing pigs and no clinical signs (increase in abortion rate, early farrowing and sudden increase in lost piglets) compatible with a PRRSV outbreak were observed. In conclusion, both farms was classified as PRRSV positive stable^16^, which means that PRRSV was endemically circulating probably during the growing period in the animals.

There is only data of one year of production for the former two farms. In both farms, it was observed cases of complex porcine respiratory disease in many batches with variable levels of affection (increase of mortality, cough, growth retardation and increase of substandard pigs). The mortality during the lactation, nursery and fattening period was 12.1, 4.5 and 3.5 for the control versus 12.3, 3.5 and 2.5 in the experimental group, respectively in the farm 1 (25,000 pig in each group approximately). In the case of farm 2, the mortality during the lactation, nursery and fattening period was 11.9, 5 and 4.5 for the control versus 11.8, 4 and 3.5 in the experimental group, respectively (29,000 pig in each group approximately). In both farms, the global mortality (nursery plus fattening period) for the control group during the rearing period was significantly higher than for the experimental one.

## Discussion

This study developed a new simulation model designed to mimic the intra-herd dynamics of a typical pig production system in which the effects of management measures are reflected in the control of the PRRSV under several epidemiologic scenarios. Results suggest that no cross-fostering (NCF) during lactation and the maintaining of litter integrity (LI) significantly decrease the number of sick and dead animals during the rearing period compared to scenarios where cross-fostering (CF) and no litter integrity (NLI) are practiced, regardless of the percentage (1–10%) of PRRSV infected sows. These results highlight the relevance of reducing cross-fostering and maintaining litter integrity as techniques for controlling PRRSV and quantifying its effects. The results predicted by the model have been validated in two real farms registering mortality during the lactation, nursery and fattening as key parameters of pig production.

Knowledge of PRRSV epidemiology and control measures must be continuously refined and updated. The use of mathematical modelling is becoming increasingly common in situations in which traditional epidemiological studies are not feasible due to particular field conditions. These models allow assessing control strategies in cases in which traditional experiments are impractical. Mathematical models can be classified as deterministic or stochastic, with the relevant difference that stochastics models are able to embrace the variability between individuals and to incorporate chance. They have previously been used in PRRSV epidemiology modelling to describe the dynamics of this type of viral infection in pig populations^12,17,18^. Recently, a new discrete event, agent-based model has been proposed for PRRSV epidemiology modelling^19^ that allows for biological variability between individuals^20^. Moreover, this model allows modelling and updating the relationship between agents as animals move through different locations and stages of production. In the present study, we used population dynamic P models (PDP models) to assess the potential impact of different management scenarios on PRRSV epidemiology and its clinical consequences. These models mimic the functioning of cells and are able to emulate flows in pigs and allow for the incorporation of stochasticity into the PRRSV parameters that are subject to variability and uncertainty. Parameters used for modelling the transmission within and between populations were primarily obtained from the bibliography^12,19,21^. However, as occurs in any model, some limitations arise. For example, we are using the same reproduction ratio (R_0_) value from lactation to fattening and it is highly improbable that this value would be identical taking into account the role of colostral antibodies in neonatal piglets and the change of susceptibility to PRRSV with age, but there is no published information about PRRSV transmission neither during the lactation period nor during all the range of pig ages. Thus we have believed more reasonable to maintain a R_0_ value of 5 close to the maximum value published^17,22–25^ across all the production phases for genotype 1 PRRSV strains instead of change this value arbitrarily without robust data. On the other hand, a strength of the model is based on the possibility to work under different scenarios (herd size, virus virulence, different contact patterns, and disease and production parameters) for which different preventive medicine strategies (e.g. vaccination) could be applied. According to the literature, this is the first time that this modeling approach has been applied to swine epidemiology, although it has been previously used in ecological processes^26–28^.

Internal biosecurity is a widely accepted method for addressing a variety of infectious problems^29^. This approach aims to decrease the spread of infectious agents through populations. For instance, the indirect transmission rate was found to be approximately 10 times lower than the direct rate for *Actinobacillus pleuropneumoniae*^30^. A similar value has also been found for *Streptococcus suis*^31^ and viral pathogens (e.g. classical swine fever virus^32^, foot-and-mouth disease virus^33^, and porcine circovirus (PCV2)^34^. In the case of PRRSV, the value for the basic reproduction number (*R*_0_) is quite low (3–5), at least for European field strains^21^, which suggests that the control of PRRSV transmission by separating uninfected and infected pigs may help reduce prevalence under field conditions. However, the available data is insufficient to obtain robust population-based estimates of efficacy since it is extremely difficult to carry out specific experiments that test management procedures. In this sense, our approach could provide valuable information for estimating the efficacy of these procedures.

This model shows that the percentage of infected and dead animals resulting from PRRSV infection decreases if cross-fostering is not carried out and litter integrity is maintained during rearing. Surprisingly, the mortality was on average relatively small for the different scenarios examined. A possible explanation could be related to the low lethality assumed in the model due to the absence of bacterial coinfections. It is obvious that this possibility could be considered exceptional because viral and bacterial infections associated with the Porcine Respiratory Disease Complex are commonly observed under field conditions^35^. Thus, in terms of mortality our findings could reflect an optimistic best-case scenario in terms of losses due to PRRSV infection. Moreover, the mortality in fatteners cannot only be attributed due to PRRSV infection but this viral infection was probably playing a major role as key component of the porcine respiratory disease complex^35^. Thus, bacterial secondary infections and mortality can be produced during the acute phase of PRRSV infection and in recovered animals but developing chronic lesions. Thus, it was considered reasonable to associate the total mortality during the fattening period to the presence of PRRSV infection even taking into account that this viral infection usually took place at the beginning of the fattening period.

The results obtained with this PDP model has been validated in two PRRSV positive stable sow farms. Thus, it was possible to validate our model in a worst-case scenario because the prevalence of this virus in piglets at weaning was probably low in these farms. The mortality observed during the nursery and fattening period was higher than those predicted in the model probably due the presence of other coinfections that our model is not tackled. In any case, the model was able to detect significant differences for production parameters between groups with different management procedures as it was also observed under field conditions. On the other hand, the model was able to estimate the percentage of infected animals at the end of the nursery period and fattening under different management strategies. In general terms, the lowest values were obtained after limiting cross-fostering and maintaining litter integrity during rearing. These results agree with other studies on the dispersion of *A. pleuropneumoniae* after the simulation of mixing litters at weaning^30^. Thus, with an initial proportion of 0.25 infected pigs, the mixing of litters would result in an extremely high prevalence of *A. pleuropneumoniae* at the end of the nursery period due probably to an increase in the within-pen transmission of this bacteria in comparison with nurseries where litter integrity is maintained after weaning^30^.

Our findings support piglet-level control strategies. One of the best known strategies is Management Changes to Reduce Exposure to Bacteria and Eliminate Losses (McRebelTM)^36^. This technique is based on limiting cross-fostering and avoiding mixing animals from different litters. McRebelTM has also been very useful in decreasing the odds of a piglet contracting Meticillin Resistant Staphylococcus Aureus (MRSA) during lactation and post-weaning^37^ and in controlling African Swine Fever Virus by boosting biosecurity measures in the event of a suspicion of infection^13^. This approach is also useful for *Mycoplasma hyopneumoniae* control because, in addition to factors such as differences in housing, the *M. hyopneumoniae* (Mhyo) strain and the type of production system, the initial prevalence of colonization in the population is crucial for defining the clinical course of the disease. Thus, a prevalence of Mhyo at weaning can be affected by the McRebel technique^38^. Finally, this approach is also useful for parasitic diseases and, for example, if cross-fostering was either not applied or was carried out only during the first 24 hours of life in a herd decrease the possibility of *Isospora suis* oocyst excretion versus herds in which the cross-fostering of piglets after day one of life was commonly carried out. Moreover, it has been observed that cross-fostered piglets may shed earlier this parasite and may be responsible for litter contamination^39^.

In conclusion, our model was able to analyze farm management procedures based on reducing cross-fostering and maintaining litter integrity to control Porcine Reproductive and Respiratory Syndrome (PRRSV) virus under different epidemiological scenarios. Thus, our results suggest that no cross-fostering during lactation and the maintaining of litter integrity significantly decrease the number of sick and dead animals during the rearing period compared to scenarios where cross-fostering and no litter integrity are practiced.

## Material and Methods

### Ethics statement

This research paper does not require an ethics statement due that no animals were used to carry out this research work.

### Farm structure and management procedures

**Figure 2:**
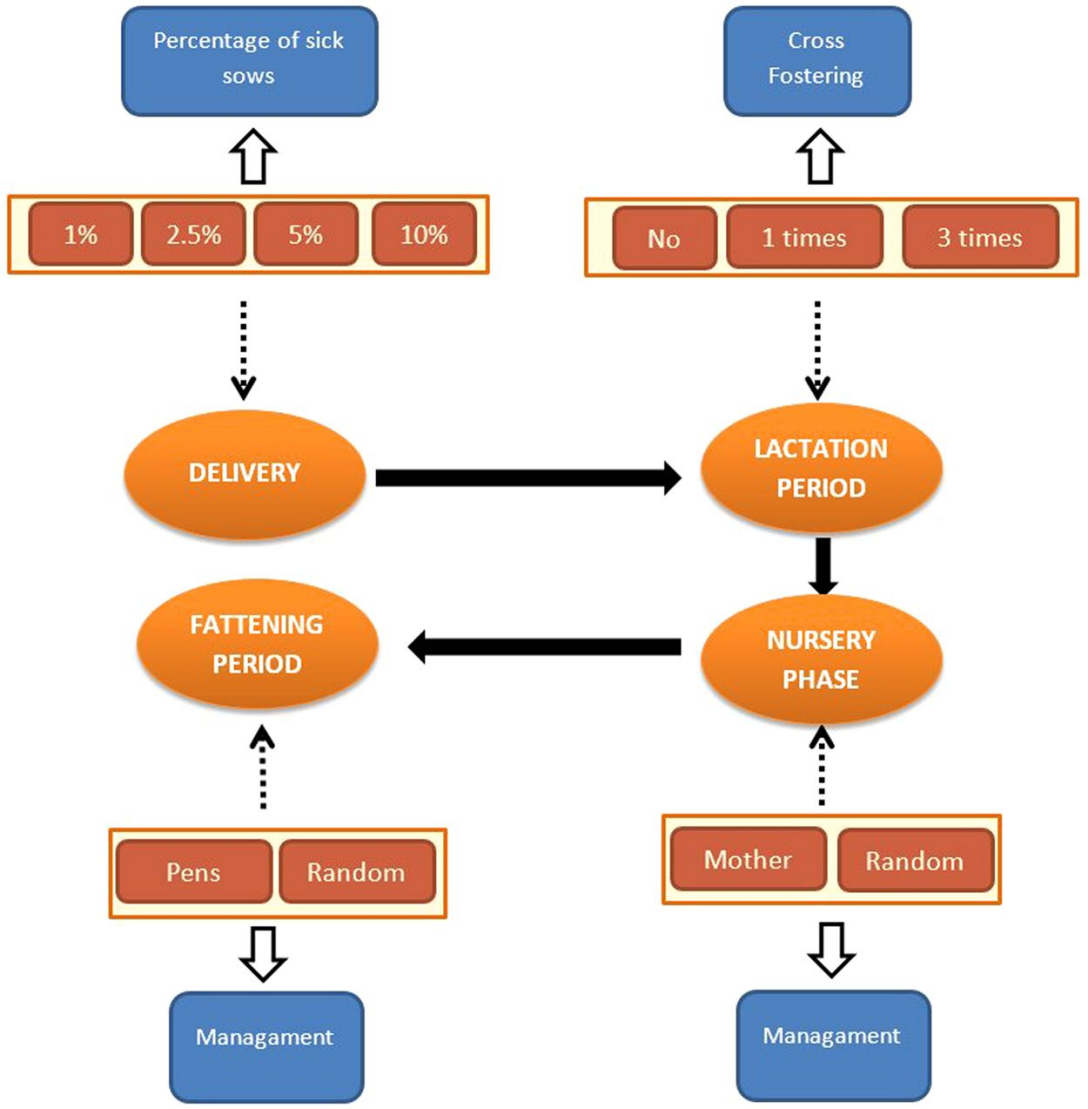
General functioning diagram of a pig farm specifying each production phase.

A stochastic PDP model for a swine breeding herd has been developed in which animals were categorized in classes according to their production stage. This model describes the spatial organization of a common swine herd and takes into account all production stages (sows, piglets and pigs) (Fig. 2). The spatial subpopulations of the model match a breeding/gestation unit and a farrowing unit where lactation takes place. In the breeding/gestation unit, sows are bred and gestate for 16 weeks. Then, they are moved to the farrowing unit where the delivery takes place during the first week (week 1) and remain until their piglets are weaned after three weeks. Weaned sows return to the breeding/gestation area where they are either culled or rebred. The nursery phase runs from 3 to 9 weeks of age and the fattening phase is the final rearing phase (from 9 to 24 weeks of age). Movement of animals between stages and sub-populations takes place on a weekly basis. Animals are assumed to mingle homogenously within their respective production units.

**Figure 3:**
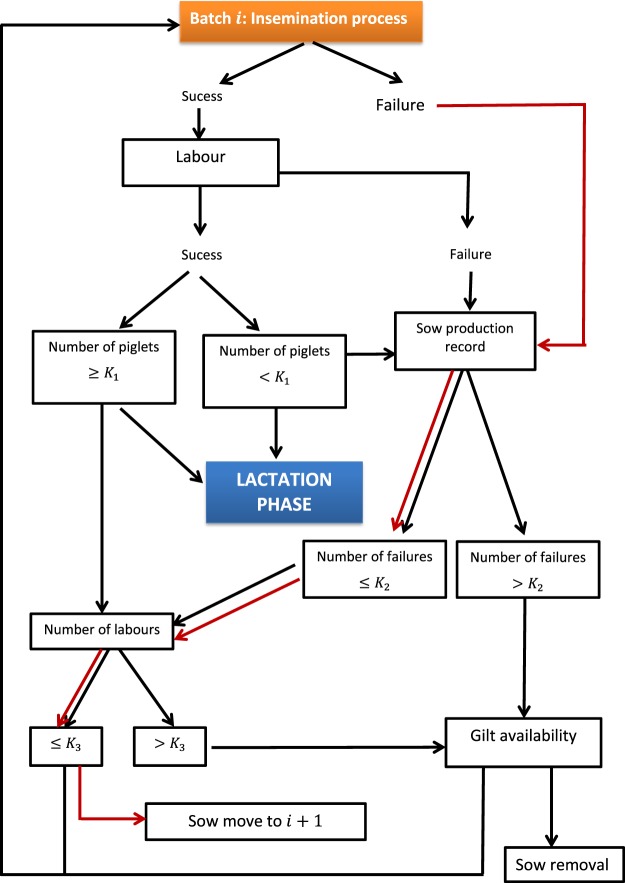
Functioning diagram of the reproductive phase in a pig farm.

In each of the production phases (reproduction-lactation, nursery and fattening) a series of management decisions is taken based on the evolution and physiological state of the animals. In phase 1, the sows are bred by artificial insemination; if the insemination is not successful, the sow’s life history is analyzed and it is decided whether it should continue its productive life or be sent to the slaughterhouse. If the sow becomes pregnant but gives birth to a number of piglets below a certain threshold, its life history is analyzed to decide its future. Depending on the number of births and the number of available productive mothers, the sow is either slaughtered or given another chance (Fig. 3).

**Figure 4:**
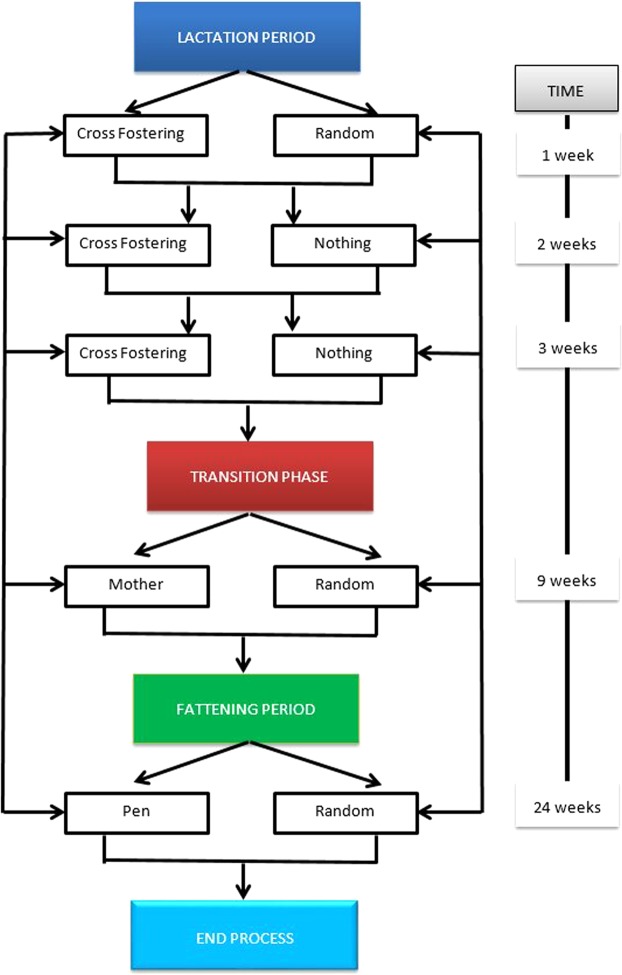
Functioning diagram of a pig farm specifying the management applied during the lactation, nursery (transition) and fattening phase.

All management decisions regarding the mixing of animals that are adopted during the rearing period are represented in Fig. 3. From a management point of view, cross-fostering can either be carried out or not during lactation; if so, it could be carried out one, two or three times. After lactation, the transition phase begins whereby piglets are separated from their mothers and are placed in pens according to one of two management strategies. One consists of grouping pigs according to the origin of their mothers and the other is based in allocating the piglets randomly by pen. In both cases, young pigs are separated according to sex. During fattening, animals are moved to other pens located in other buildings. Transfers are carried out either in an orderly manner, that is, by partially maintaining the groupings established in the previous phase, or at random (Fig. 4).

During lactation, three types of management were studied: i) not performing cross-fostering, ii) performing cross-fostering only once after delivery, and iii) performing cross-fostering three times (once a week). Extreme cases of management during transition and fattening were studied: i) orderly movements consisting of filling the pens during transition according to the sow’s origin and during fattening according to nursery distribution (MP) or ii) random movements consisting of filling the nursery and fattening pens at random (RR).

### PRRSV epidemiological scenarios

We apply bioinspired models (PDP Systems, see below) to assess the economic and animal production effects of PRRSV virus in a farm. Given that the percentage of sows positive to the PRRSV virus which are able to transmit it to their offspring is a crucial factor in the epidemiology of the PRRSV virus^12^, four situations arise that can be classified on a gradient from endemic (1% of infected females) to epidemic (10% of infected females) scenarios, passing through two intermediate situations of 2.5 and 5% infected females. If we combine these four levels of prevalence with the three levels of management during lactation, twelve starting scenarios are defined during transition.

### Population Dynamic P-system (PDP models) model

PDP models are computational models inspired by cell functioning whereby small organelles grow, evolve, reproduce, and die, meanwhile interacting with the medium and other organelles of the same or different types^40^. Cells evolve in a random way and the model PDPs imitating their functioning have probabilities associated with their rules of evolution that make them stochastic models.

**Figure 5:**
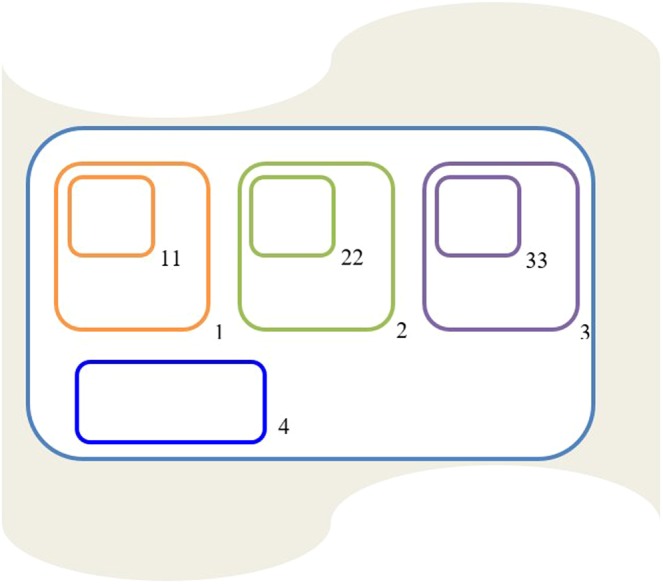
Structure of the membranes used in the population dynamic P system model designed for a pig farm.

The components of a PDP model^15^ include (1) the number of environments; (2) the membrane structure of the cell contained in each environment (Fig. 5); (3) the objects of the initial configuration; (4) the rules of evolution.

The swine farm to be modeled can have a maximum of 20 batches that are temporarily sequenced to maximize facilities and distribute production evenly throughout the year. Given that each batch is located at a different point in the production process, we decided to define an environment by batch. The covering of the sows and births will always be carried out in the same environment (environment 0); once the mothers of batch *i*, 1 ≤ *i* ≤ 20, have given birth, new objects associated with the piglets are generated. Both the objects associated with the mothers and the piglets move to environment *i* to start lactation and then continue until the animal is slaughtered. Therefore, the proposed model is composed of 21 environments.

In each environment,*e*_*i*_, 1 ≤ *i* ≤ 20, there is a cell in which lactation, transition, and fattening are sequentially simulated. To improve the operation of the model and avoid inconsistencies, four separate spaces (Fig. 5) delimited by four membranes are established within the cell defined by the skin (outer) membrane. Membrane 1, 2 and 3 were used for the lactation, nursery (transition) and fattening process, respectively whereas membrane 4 was used to stored objects and set up the initial configuration. Membranes can be either positive or negative (+, −,0) but initially their charge is always neutral (0).

The objects that appear in the initial configuration essentially consist of the physical input of the model (i.e. sows). Each sow is associated with an object,*X*_*i,j*_, with two subscripts, the first corresponding to the times it has been inseminated and the second to her number of reproductive failures. In the initial configuration, there are also other objects that are used to count steps and therefore synchronize and control the model. The objects evolve just as the animals on a farm done. The transformation of objects is performed following the rules of evolution that provide the point of departure for the model and the paths that are followed by the sows and, subsequently, by each piglet until it is sacrificed. When there are specific conditions (e.g. location or environment) the rules allow objects to evolve, to transform (e.g. movement, growth, contagious diseases), generate new objects (birth), or dissolve (die). The rules are expressed as follows:

$$r\equiv {[a,b]}_{i}^{0}\mathop{\to }\limits^{p}c{[\,]}_{i}^{+}$$

In this example, if objects *a* and *b* are in membrane *i* with charge 0, and with a probability *p*, they will evolve to object *c* and change the charge of membrane *i* to +.

The details of the rules of evolution of the model are provided in the Supporting Information. The model was run 30 times for a farm with 1000 sows and its production and epidemiological parameters (Tables 5, 6) were obtained from the literature^12,19,21–25,41^. In particular, the reproduction rate value (5) was selected based on a high value from the range described in the literature to develop a worst-case scenario (Table 7).

**Table 5:** Production parameters used to run the model in a 1000 sow farm.

Mothers	Batches	20
	Number sows per batch	50
	Fertility to delivery	0.85
	Fertility at gestation control	0.9
	Abortion probability	0.02
	Average number of live offspring	12.55
	Average number of stillborn piglets	1.5
	Sow mortality	0.06
	Maximum number of deliveries by mother	8
	Maximum number of failures by mother	3
	Minimum number of failures by mother to remove	2
	Maxim parity to be removed	5
	Annual sow replacement rate	0.49
Lactation period Piglet data	Mortality week 1	0.06
	Mortality week 2	0.04
	Mortality week 3	0.02
Transition period	Number of pen	22
	Mortality	0.03
Fattening period	Number of pen	44
	Mortality	0.04

**Table 6:** Epidemiological parameters used to run the model in a 1000 sow farm.

Percentage of infected sows (%)	1%, 2.5%, 5% and 10%		
Probability to transmit the virus from sow to piglets	0.728		
	**Lactation period**	**Nursery**	**Fattening**
Reproduction rate (*R*_0_)	5	5	5
Infectious period (days)	42	42	42
Lethality	0.1	0.1	0.1

**Table 7:** Available literature to select the reproduction rate (R_0_) as a key epidemiological parameter in this research work.

Age and animals and conditions	*R*_0_(Confidence interval 95%)	Bibliographic reference
Nursery piglets (five weeks of age) and experimental conditions with genotype 1 PRRSV strains	2-infinite	23
Nursery piglets (five weeks of age) and experimental conditions with genotype 1 PRRSV strains	2.6 (2.1–3.4)	22
Nursery piglets (seven weeks of age) mimicking natural conditions with genotype 1 PRRSV strains	5,4 (2.9–9)	25
Nursery piglets (nine weeks of age) mimicking natural conditions with genotype 1 PRRSV strains	2.78 (2.1–3.4)	21
Endemic farms Longitudinal study	Farm 1: 3.5 Farm 2: 5.5	24

### Uncertainty and sensitivity analyses

The randomness and complexity of the model suggests that the variability in the final results does not depend on a single parameter, thereby making it difficult to study the sensitivity of the model in relation to all the different values of the parameters (see Supporting Information). However, one way to study the effect of all parameters simultaneously is to find the response surface of the model using an independent quadratic design such as the Box-Behnken design. These response surface designs are used to model the relationship between the independent variables or factors and the response variable^42^, using in this case quadratic models.

Using the pig PDP model, the necessary virtual experiments (28 experiments) were carried out for the Box-Behnken design of four factors at two levels (Table 8). The factors studied were: percentage of infected sows, probability of transmission of the disease from mothers to piglets, the *R*_0_ value, and the lethality of the disease. There is a range of variability for each of the factors (the extremes of the intervals are the two levels of the design) (Table 8). The percentage of infected animals at the end of the lactation, nursery and the fattening process were considered as the response variables.

**Table 8:** Range of values for the percentage of infected sows, the probability of disease transmission during the lactation phase, reproduction rate (*R*_0_) and disease lethality to carry out the uncertainty and sensitivity analysis.

Parameter	Low Level	High Level
Infected sow	0.01	0.1
Probability of disease transmission during the lactation phase	0.6	0.9
*R*_0_	3	8
Disease lethality	0.05	0.2

Our goals were to test whether the management carried out during the rearing period affects the final result calculated as i) the proportion of infected animals at the end of nursery (transition) and fattening phase and ii) the number of animals infected during the rearing cycle, and iii) the number of animals that died in the nursery and fattening period. The independent variables considered were:

- Proportion of infected mothers during lactation: 1%, 2.5%, 5%, or 10%.
- Management performed during lactation: randomization of piglets, cross-fostering during the first week, and cross-fostering during the 3 weeks of lactation.
- Management during the nursery period: management by mothers, M, and random, R.
- Fattening phase management: management by pen (according to the nursery phase), P, and random management, R.

Combining the possible management techniques during the two final phases (nursery and fattening period), four scenarios were obtained: MP, MR, RP, and RR. The most extreme cases were studied: the most conservative case from the point of view of disease transmission (MP) and the case that theoretically favors most disease transmission (RR).

In order to avoid concealing results that were due to the management performed during nursery and fattening, simulations were carried out for the 12 possible scenarios obtained with the four levels of infected sows and the three types of management during lactation, the model being halted before the beginning of the nursery phase. These results were used as the starting point for the simulations conducted to study the effect of management during the final two phases.

#### Validation with farm data

Pig production records are being registered to validate this PDP model in ten farms of 2000 sows approximately during one year. PRRSV health status for these farms was monitorized following previously published recommendations^16^. Briefly, herd classification for PRRSV was based on determining both shedding and exposure status of the herd. Quantitative reverse transcriptase PCR (qRT-PCR) and antibody testing are methods to determine shedding and exposure to this virus, respectively. Details of these techniques have been previously published^43^. Classification was established by monitoring the PRRSV status of specific subpopulations in a herd. In our case, the studied subpopulations were adult breeding animals, weaning-age pigs and growing pigs (24 weeks of age). Testing was based upon monthly sampling of 30 and 60 serum samples for shedding and exposure, respectively, from each animal subpopulation as clearly set up in the literature^16^. This sampling strategy allows detecting the presence of the virus with prevalence equal or higher than 10% and with a confidence level of 95%. The PRRS virus shedding status was classified as negative or positive. A negative shedding status meant that all the serum samples tested by qRT-PCR were negative (absence of viral shedding in the herd) while a positive shedding status meant that, at least, one serum sample tested positive by qRT-PCR (evidence of viral shedding and transmission in the herd). The exposure status was also classified as negative or positive. Thus, a negative exposure status meant there was absence of antibodies to the virus in the samples tested. On the contrary, a positive exposure meant that there was presence of antibodies to the virus. Finally, the health status of the farm was considered endemic (EN) or epidemic (EP) according to the absence or presence of overt reproductive problems in the sow farm, respectively. This overt reproductive problems (epidemic situation) were based on a significant increase of abortions and/or lost piglets (stillborns and mummified) versus the baseline situation (endemic situation) in the sow farm.

The pigs of each batch were split in two flows that were defined as control and experimental, and that were allocated in different facilities but inside the same farm. In the control group, cross-fostering was performed once after delivery in the lactation phase and pigs filled randomly the nursery and fattening phase whereas, in the experimental group, no cross-fostering was carried out during the lactation phase and orderly movements consisting of filling the pens during transition according to the sow’s origin and during fattening according to nursery distribution. It was registered the mortality during the lactation, nursery and fattening phase because these parameters can be easily and accurately recorded under field conditions. Until now, there is only data of two farms that can be considered as a proof of concept.

### Statistical analyses

The results were analyzed using a linear ANOVA model. The design variables were: (1) proportion of infected mothers, (2) management during lactation (existence of cross-fostering), and (3) management during nursery and fattening. The response variables correspond to the number of infected animals at the end of nursery and fattening period, the number of animals infected during the rearing cycle and the number of animals that died in the nursery and during fattening. The effect is considered to be significant when p < 0.05.

## Supplementary information

Supporting Information
