# Supplementary material for: Improving the management procedures in farms infected with the Porcine Reproductive and Respiratory Syndrome virus using PDP models
Source: Sci Rep. 2019 Jul 10;9:9959. doi: 10.1038/s41598-019-46339-w (PMC6620323; doi:10.1038/s41598-019-46339-w)
Supplement: Supplementary file 1 — Supporting Information [file 41598_2019_46339_MOESM1_ESM.pdf]

Supporting information for:

Improving the management procedures in farms infected with the Porcine and Respiratory Syndrome virus using PDP models

Mª Àngels Colomer, Antoni Margalida, Lorenzo Fraile

Uncertainty and sensitivity analyses

The uncertainty and sensitivity of the model was studied through the response surface calculated, using a Box-Bhenken design, using as factors: Percentage of infected sows (*MI*), the probability of transmission during the lactation phase (*PT*), the basic reproduction rate (R_0) during the nursery and fattening period (it is used to measure the transmission potential of a disease) and lethality of the disease (L). For each factor, it is needed a range of values from low to high (Table 2). The response variables were the percentage of sick animals at the end of the lactation (*L*), transition (*T*) and fattening phase (*F*).

The Box-Bhenken design of 4 factors with two levels was carried out by 28 experiences that have been simulated using the PDP model. The three response surfaces are:

Percentage of sick animals at the end of the lactation phase:

$$P_{FL} = 0.0492 - 0.0116 \cdot R_0 - 0.0092 \cdot MI \cdot R_0 + 0.0036 \cdot PT \cdot L + 0.0492 \cdot L^2$$

Percentage of sick animals at the end of the transition phase:

$$P_T = 0.2421 + 0.1669 \cdot MI + 0.0993 \cdot R_0 - 0.0322 \cdot L + 0.0645\ MI \cdot R_0 - 0.0227\ MI \cdot L - 0.0269 \cdot MI^2$$

Percentage of sick animals at the end of the fattening phase:

$P_F = 0.2177 + 0.1408 \cdot MI + 0.1106 \cdot R_0 - 0.0229 \cdot L + 0.0677 \cdot MI \cdot R_0 - 0.0323 \cdot MI^2$

The results using this Box-Bhenken design previously described are detailed in the following table:

	End lactation phase		End transition phase		End fattening phase	
	Value	P-value	Value	P-value	Value	P-value
Intercept	0.0492	< 2.2e-16 ***	0.2421	2.204e-15 ***	0.2177	5.454e-13 ***
MI	0.0003	0.767	0.1669	2.238e-15 ***	0.1408	1.267e-13 ***
PT	0.0012	0.216	-0.0030	0.456	-0.0039	0.404
Ro	-0.0116	1.026e-08 ***	0.0993	1.767e-12 ***	0.1106	2.796e-12 ***
L	0.0019	0.243	-0.0322	1.557e-06 ***	-0.0229	0.0002 ***
MIxPT	0.0018	0.287	0.0078	0.269	0.0070	0.386
MIxRo	-0.0092	5.614e-05 ***	0.0645	3.056e-07 ***	0.0677	9.037e-07 ***
MIxL	-0.0034	0.051	-0.0227	0.0052**	-0.0134	0.109
PTxRo	-0.0016	0.321	-0.0115	0.112	-0.0102	0.214
PTxL	0.0036	0.041 *	-0.0029	0.679	-0.0013	0.868
RoxL	-0.0012	0.384	-0.0070	0.319	-0.0120	0.149
MI^2	0.0001	0.967	-0.0269	0.0003 ***	-0.0323	0.0002 ***
PT^2	0.0001	0.944	0.0025	0.653	0.0033	0.618
Ro^2	0.0008	0.568	0.0058	0.309	0.0070	0.291
L^2	0.0492	< 2.2e-16 ***	0.0025	0.661	0.0013	0.839

The percentage of infected sows, R_0 and lethality are the parameters that have a significant effect on the percentage of sick animals at the end of the nursery and

fattening period. However, the probability of transmission of the disease during the lactation phase slightly affects during this phase.

Using the response surfaces, sensitivity has been studied in the three phases (*L*: lactation, T: transition and *F*: fattening of the pig) (Figures 1-3).

Figure S1. Variation in the percentage of sick animals depending on the percentage of infected mothers in the lactation (L), nursery (T) and fattening period (F). Green, blue and red colour represents high, intermediate and low level, respectively for the other parameters included in these analysis.

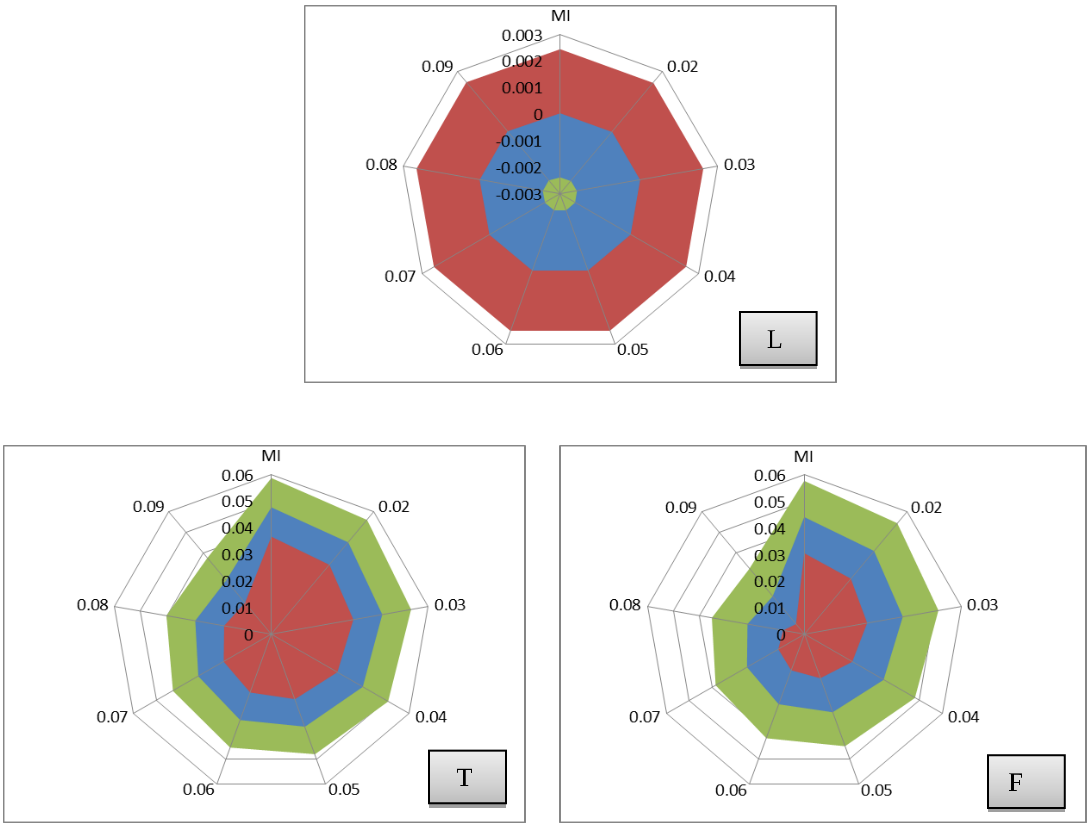

The increase of infected piglets is very small during the lactation phase in the range of values used for infected sows. However, in the transition and fattening phase, this increase is bigger using low versus high level of infected sows. During the rearing period (transition and fattening), an increase of one point (1%) in the percentage of infected sows implied an increase between 3 and 6 % of infected pigs.

Figure S2. Variation in the percentage of sick animals, as a function of the R_0, value in the lactation (L), nursery (T) and fattening (F) period. Green, blue and red colour represents high, intermediate and low level, respectively for the other parameters included in these analysis.

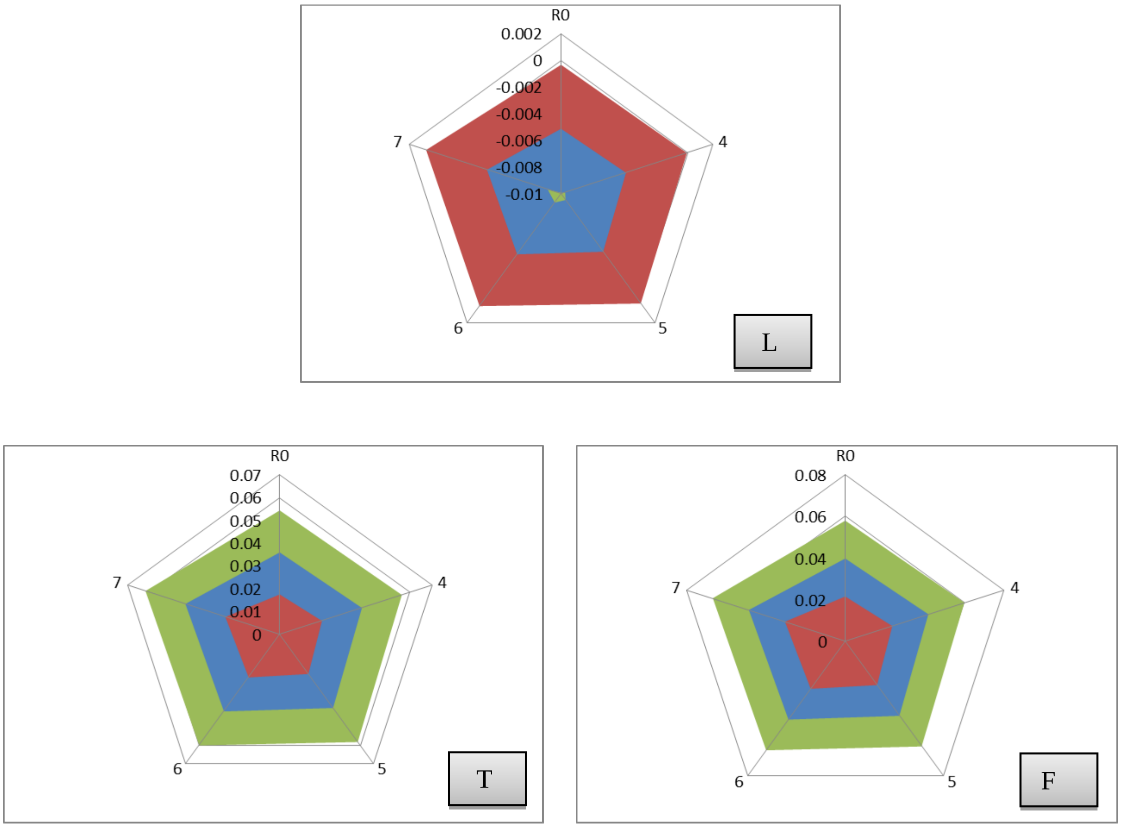

In the case of the R_0 parameter, an increase of one unit implies similar variations (between 2 to 5%) in the percentage of sick animals during the nursery and fattening phase in the range of studied values. However, in the lactation phase, this increase is practically nil.

Figure S3. Variation in the percentage of sick animals as a function of the lethality in the lactation (L), nursery (T) and fattening (F) period. Green, blue and red colour represents high, intermediate and low level, respectively for the other parameters included in these analysis.

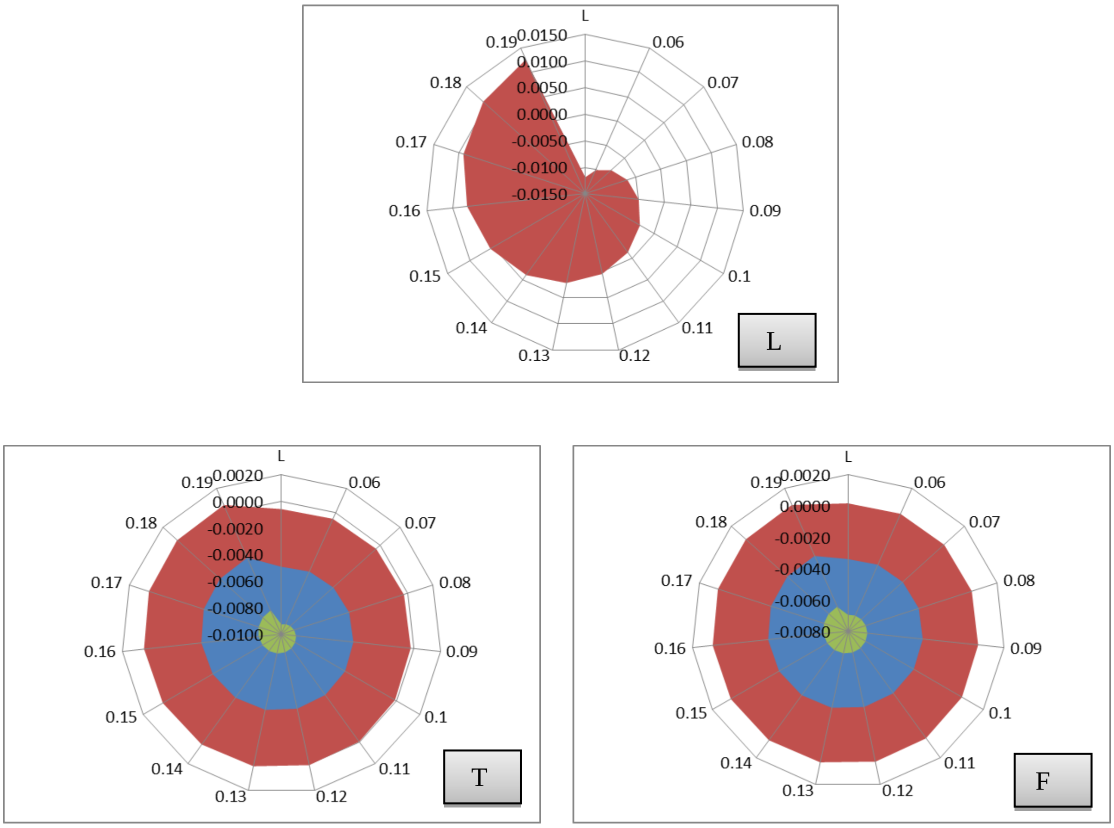

In the case of variations in the lethality value, the increase in sick animals does not practically vary during the transition and fattening period in the range of studied values. In the lactation phase, it increases as the lethality increases, but the variations are very small.

Pig PDP model

A PDP model is formed by:

1. Number of environment
2. Membrane structure
3. Initial configuration
4. Evolution rules

Number of environment

There is an environment for the delivery phase (environment 0 is labelled by 100) and 20 environments for the rest of the phases, one per batch (labelled by 100 + batch).

Membrane structure

$$\mu = [[[\quad]_{11}]_1[[\quad]_{22}]_2[[\quad]_{33}]_3[\quad]_4]_0$$

In all environments there is a cell with the same structure.

Initial configuration

The processes performed in environment 0 are different from other environments, so the initial configuration will be also different.

Environment 0

$\mu_0 = \left\{X_{i,k,F}^{n_{i,k,F}}, 1 \leq i \leq bloc, 0 \leq k \leq partos, 0 \leq F < maxfrac;\ D_1; RM_0; M'_j, 1 \leq j \leq q; Bc_i, 1 \leq i \leq bloc\right\}$

$\mu_1 = \{cycle_1\}$

Each mother is associated with an object $X_{i,k,F}^{n_{i,k,F}}$, the indexes i,k and F indicate respectively the batch to which the mother belongs, the number of births she has performed and the number of births failed, either by having aborted or by having few live pigs.

D_1 allows selecting the mothers that belong to batch 1, this object evolves and at the end of the process for the mothers of the first batch has been transformed to D_2 that will allow to select the mothers of the next batch and so on until arriving at batch number 20. The object D_{20} will evolve to object D_1, starting the process again.

RM_0 It is a counter that facilitates the synchronization of the model.

M'_j, objects associated with the mothers that are going to give birth, this object allows to simulate if the mother has successfully raised or not and the number of live piglets she has.

Bc_i object that is used to ensure that the start warning of the phase 2 processes of the model reaches all the environments associated with each batch.

$cycle_1$ allows to control the cycle that is being carried out and the number of times that the mothers of each batch are covered in the year.

$Environment\ \ k\ (1 \leq k \leq bloc)$

$$\mu_0 = \left\{MT_i, 1 \leq i \leq q;\ PN_i, 1 \leq i \leq floor\left(\frac{Tpen}{2}\right);\ PNE_i, 1 \leq i \leq Epen\right\}$$

In the transition phase the males will be separated from the females, half of the pens will be for each sex. Half of the pens of the transition phase are associated with an object PN_i and all the pens of the phase of fatering an object PNE_i. The object MT_i, is associated with the mother, this object will be used in the transition phase.

The parameters used in the model are shown on Table S1.

Table S1 Parameters of the model.

MOTHERS	Definition	Value
q	Blocks	20
$bloc$	Number sows to give birth at a time	50
f'	Fertility to delivery	0.85
f	Fertility at 21 days	0.9
abt	Probability abortion	0.02
Avl	Average number of live offspring	12.55
Avd	Average number of stillborn pigs	1.5
mm	Natural mortality adults	0.06
$partos$	Maximum number of births by mother	8
$maxfrac$	Maximum number of failures by mother	3
$minfrac$	Minimum number of failures by mother to remove	2
$minpig$	Minimum number of offspring not to be eliminated	5
rv	Percentage mothers who are renewed by cycle	0.49
$lvp\{i\}$	Random values Poisson distribution parameter $Avl\{1\}$. $1 \leq i \leq 100$	
$lv\{i\}$	$Min\{lvp\{i\}, 20\}$, $1 \leq i \leq 100$	
$ld\{i]$	random values of a Poisson distribution of parameter Avd. $1 \leq i \leq 100$	
$lpigs\{i\}$	$minpig - Id\{i\} - lv\{i\}$, $1 \leq i \leq 100$	
$Faultp\{i\}$	returns 1 if $lpigs\{i\} >\ 0$; 0 otherwise , $1 \leq i \leq 100$	
$Fault\{i\}$	Return floor $Faultp\{i\}$, $1 \leq i \leq 100$	
mi	Percentage of infected mothers.	0.1*
li	Probability to transmit to pigs the disease	0.728
PIGLETS		
$ml\{1\}$	Mortality week 1.	0.06
$ml\{2\}$	Mortality week 2.	0.04
$ml\{3\}$	Mortality week 3.	0.02
$Avl\{i+1\}$	$Avl\{i\} \cdot (1 - ml\{i\}), 1 \leq i \leq 2$	
$AVLweight$	Average weight at the end of the period (gr)	6480
$SDLweight$	Deviation weight at the end of the period (gr)	1000
$loss$	Weight loss for cross fostering (gr)	100
gm	Equal 1 random management, otherwise equal 0	
$WeekCross\{$[illegible]	Equal to 1 if crosfostering is done, 0 otherwise	
RL	Rate of reproduction of infected animals	5
DL	Reproductive rate days that take to become infected	42
$IncL$	Incubation period (days)	7
$InfL$	Infective period (days)	42
LL	Lethality (probability)	0.1
VL	Percentage of vaccinated animals	0
EVL	Vaccine effectiveness	0.7
ctg	Contagious disease equal 1, otherwise 0.	1
$lossdiseadeL$	Reduction of weight at 21 days (gr)	100

KL	$INT\left(\frac{RL\cdot 7}{42}+0.5\right)$	
pL	$1-VL\cdot EVL$	
$InfL$	$MIN(21, InfL)$	
NURSERY		
$Tpen$	Number of pen	22
mt	Natural mortality	0.03
$AVTweight$	Average weight at the end of the period (gr)	2000<br>0
$SDTweight$	Desviation weight at the end of the period (Kgr)	2000
N	$5\cdot Tpen+1$	
$Lweight\{i\}$	Random values Normal distribution of parameter $AVLweight$, $SDLweight, 1\le i\le N$	
$Tweight\{i\}$	Random values Normal distribution of parameter $AVTweight$, $SDTweight, 1\le i\le N$	
$gt\{i\}$	$gt\{1\}=1$ distribution by mothers; $gt\{2\}=1$ random distribution	
RT	Rate of reproduction of infected animals	5
DT	Reproductive rate days that take to become infected	42
$IncT$	Incubation period (days)	7
$InfT$	Infective period (days)	42
LT	Lethality (probability)	0.1
VT	Percentage of vaccinated animals	0
EVT	Vaccine effectiveness	0.7
ctg	Contagious disease	1
$lossdiseadeT$	Weight loss (gr)	500
$avIncfInft$	$INT\left(\frac{IncT+InfT}{2}\right)$	
$avInc$	$INT\left(\frac{IncT}{2}\right)$	
KT	$INT\left(\frac{RT\cdot(1-VT\cdot EVT)}{DT}\right)$	
pT	$\frac{RT\cdot(1-VT\cdot EVT)}{DT\cdot KT}$	
FATTENIN<br>G		
$Epen$	Number of pen	44
me	Natural mortality	0.04
$AVEweight$	Average weight at the end of the period (kg)	10
$SDEweight$	Deviation weight at the end of the period (kg)	1
$N1$	$5\cdot Epen+1$	
$Eweight\{i\}$	Random values Normal distribution of parameter $AVEweight$, $SDEweight, 1\le i\le N1$	
$ge\{i\}$	$ge\{1\}=1$ distribution by mothers; $ge\{2\}=1$ random distribution	
RE	Rate of reproduction of infected animals	5
DE	Reproductive rate days that take to become infected	42
$IncE$	Incubation period (days)	7
$InfE$	Infective period (days)	42
LE	Lethality (probability)	0.1

VE	Percentage of vaccinated animals	0
EVE	Vaccine effectiveness	0.7
ceg	Contagious disease	1
$lossdiseadeE$	Weight loss (gr)	1000
KE	$INT\left(\frac{RE\cdot(1-VE\cdot EVE)}{DE}\right)$	
pE	$\frac{RE\cdot(1-VE\cdot EVE)}{DE\cdot KE}$	

Phase 1: Birth

Step 1

Object D_i will generate as many objects a_i as mothers have each batch (q). An object XN' is generated for each mother that must be renewed. A copy of object D' is saved, and objects d that store batch information are generated.

$$r_1 \equiv D_i[\quad]_1^0 \longrightarrow a_i^q, dd_i\ \left[XN'^{rv\cdot q}, D'_i, d_i^{q\cdot Av_1\cdot 4}\right]_1^+, 1 \le i \le bloc.$$

$$r_2 \equiv RM_0[\quad]_1^0 \longrightarrow RM_1\ [\quad]_1^+.$$

Step 2

The mothers who are going to start the reproduction process will enter the membrane 1.$r_3 \equiv X_{i,k,F}, a_i[\quad]_1^+ \longrightarrow \left[Y_{k,F}\right]_1^0, 1 \le i \le bloc, 0 \le k \le partos, 0 \le F \le maxfrac.$

Mothers who are covered and who also do not abort, generate objects S_k that indicates that they have given birth successfully, $CH_{j,k}$ that keeps information about the number of piglets and copies of the object that has evolved, M_j, M'_j.

$$r_4 \equiv M'_j[\quad]_1^+ \xrightarrow{\frac{f\cdot(1-abt)}{100}} \left[S_k, CH_{j,k}, M_j, M'_j\right]_1^0, 1 \le j \le q, 1 \le k \le 100.$$

Mothers who have not given birth successfully generate objects S' and a copy of the object M'_{j}. The M_{j} object will only exist for mothers who have given birth successfully.

$$r_5 \equiv M'_j[\quad]_1^+ \xrightarrow{1-f\cdot(1-abt)} \left[S', M'_j\right]_1^0, 1 \le j \le q.$$

Evolution of the objects associated with the mothers that must be renewed.

$$r_6 \equiv [XN']_1^+ \longrightarrow [XN]_1^0.$$

Evolution of the object D'_i i that will be used to start the birth of the mothers of the next batch. It is necessary to have the object D'_i, because at the end of this phase the object evolves in the same membrane with positive charge.

$r_7 \equiv [D'_i]_1^+ \longrightarrow [D_i]_1^0, 1 \leq i \leq bloc.$

$r_8 \equiv RM_1[\quad]_1^+ \longrightarrow RM_2[\quad]_1^0.$

Step 3 Fertility and misbirth

Evolution of mothers who have given birth successfully. In the case that the number of piglets is small, a failure is added.

$r_9 \equiv \left[Y_{k,F}, S_i \longrightarrow Z_{k+1,F+Fault_i}\right]_1^0, 1 \leq i \leq 100, 0 \leq k \leq partos, 0 \leq F < maxfrac.$

Mothers who have not given birth are added a fault.

$r_{10} \equiv \left[Y_{k,F}, S' \longrightarrow Z'_{k,F+1}\right]_1^0, 0 \leq k \leq partos, 0 \leq F < maxfrac.$

They will generate as many LV objects as live piglets each mother has had.

$r_{11} \equiv \left[CH_{j,k} \longrightarrow LV_j^{lv_k}\right]_1^0, 1 \leq j \leq q, 1 \leq k \leq 100.$

Counter evolution

$r_{12} \equiv [RM_2 \longrightarrow RM_3]_0^0.$

Step 4 Natural mortality

It differentiates mothers who have given birth to those who have not done so successfully. When they die, the fre object is generated indicating that a free position remains and that it must be replaced by a new mother.

$r_{14} \equiv \left[Z_{k,F} \xrightarrow{1-mm} V_{k,F}\right]_1^0, 1 \leq k \leq partos + 1, 0 \leq F \leq maxfrac.$

$r_{15} \equiv \left[Z_{k,F} \xrightarrow{mm} fre\right]_1^0, 1 \leq k \leq partos + 1, 0 \leq F \leq maxfrac.$

$r_{16} \equiv \left[Z'_{k,F} \xrightarrow{1-mm} V'_{k,F}\right]_1^0, 0 \leq k \leq partos, 1 \leq F \leq maxfrac.$

$r_{17} \equiv \left[Z'_{k,F} \xrightarrow{mm} fre\right]_1^0, 0 \leq k \leq partos, 1 \leq F \leq maxfrac.$

Counter evolution

$r_{18} \equiv [RM_3 \longrightarrow RM_4]_0^0.$

Step 5 Manager: eliminate + new mothers step 1

Mothers who have reached the maximum number of births and have also had some failure during their productive life will be replaced, so they will evolve to $fre.$ objects.

$r_{19} \equiv \left[V_{k,F} \longrightarrow fre\right]_1^0, partos \leq k \leq partos + 1, 1 \leq F \leq maxfrac.$

Mothers who reach the maximum number of births have never had a failure evolve generating two objects, $U_{k,0}, free$, the first keeps the information of the mother and the second opens the possibility that the mother is replaced. In the event that there are not enough new mothers to be replaced, this mother will be kept in the batch one more birth.

$r_{20} \equiv \left[V_{k,0} \longrightarrow U_{k,0}, fre\right]_1^0, partos \leq k \leq partos + 1.$

Mothers who have reached the maximum number of failures are eliminated.

$r_{21} \equiv \left[V_{k,maxfrac} \longrightarrow fre\right]_1^0, 1 \leq k < partos.$

Mothers who have not reached the maximum number of deliveries have had a number of important failures (but have not reached the maximum to be eliminated) evolve generating the object $U_{k,F}$, in the case of missing young mothers to replace the that must be removed there is a possibility that one more delivery will remain in the system.

$r_{22} \equiv \left[V_{k,F} \longrightarrow U_{k,F}\right]_1^0, 1 \leq k < partos, minfrac \leq F < maxfrac.$

Mothers who do not reach the maximum number of births and the number of failures have not exceeded the limit value will remain in the batch.

$r_{23} \equiv \left[V_{k,F} \longrightarrow W_{k,F}\right]_1^0, 1 \leq k < partos, 0 \leq F < minfrac.$

The evolution of the objects associated with mothers who have not given birth successfully is similar to those who have given birth successfully.

$r_{24} \equiv \left[V'_{k,F} \longrightarrow fre\right]_1^0, partos \leq k \leq partos + 1, 1 \leq F \leq maxfrac.$

$r_{25} \equiv \left[V'_{k,F} \longrightarrow fre\right]_1^0, 0 \leq k < partos, minfrac < F \leq maxfrac.$

Mothers who are not eliminated are passed to the next batch.

$r_{26} \equiv \left[V'_{k,F}, d_i\right]_1^0 \longrightarrow X_{i+1,k,F}[\quad]_1^0, 1 \leq i \leq bloc, 0 \leq k < partos, 1 \leq F \leq minfrac.$

$r_{27} \equiv \left[V'_{k,F}, d_{bloc}\right]_1^0 \longrightarrow X_{1,k,F}[\quad]_1^0, 0 \leq k < partos, 1 \leq F \leq minfrac.$

Young mothers are incorporated into the batch.

$r_{28} \equiv \left[XN, fre \longrightarrow W_{0,0}\right]_1^0.$

Evolution of the counter.

$r_{29} \equiv [RM_4 \longrightarrow RM_5]_0^0.$

<u>*Step 6 Manager: new mothers step 2*</u>

In this step, there are still objects fre generated in the previous step and that have preference in the renovation that the objects $U_{k.F}$ the latter evolve to objects $U'_{k.F}$.

$r_{30} \equiv \left[U_{k,0} \longrightarrow U'_{k,0}\right]_1^0, partos \leq k \leq partos + 1.$

$r_{31} \equiv \left[U_{k,F} \longrightarrow U'_{k,F}\right]_1^0, 1 \leq k < partos, minfrac \leq F < maxfrac.$

Evolution of the counter.

$r_{32} \equiv [RM_5 \longrightarrow RM_6]_0^0.$

<u>*Step 7 Manager: new mothers*</u>

In the case that in this step there are objects fre means that there are not enough young mothers to renew all those that should be eliminated from the batch, so that they will keep the best ones that had reached the conditions to be replaced.

$r_{33} \equiv \left[U'_{k,0}, fre\right]_1^0 \longrightarrow \left[W_{k,0}\right]_1^+, partos \leq k \leq partos + 1.$

$r_{34} \equiv \left[U'_{k,F}, fre\right]_1^0 \longrightarrow \left[W_{k,F}\right]_1^+, 1 \leq k < partos, minfrac \leq F < maxfrac.$

Evolution of the counter.

$r_{35} \equiv RM_6[\quad]_1^0 \longrightarrow RM_7[\quad]_1^+.$

<u>Step 8 Preparation of mothers for a new birth</u>

The objects associated with the mothers that are going to remain in the batch, go out to the skin membrane (labeled with the value 0) prepared to start a new reproductive cycle.

$$r_{36} \equiv [W_{k,F}, d_i]_1^+ \longrightarrow X_{i,k,F}[\quad]_1^-, 1 \le i \le bloc, 0 \le k \le partos + 1, 0 \le F \le maxfrac.$$

Objects associated with potentially renewable mothers that have not evolved, $U'_{k,F}$, are eliminated (mothers who have been renewed)

$$r_{37} \equiv [U'_{k,F}]_1^+ \longrightarrow [\#]_1^-, 1 \le k \le partos + 1, 0 \le F < maxfrac.$$

In this step, if there are fre objects, or XN objects (young mothers that have not been used to replace batch mothers). These objects are eliminated.

$r_{38} \equiv [fre]_1^+ \longrightarrow [\#]_1^-.$

$r_{39} \equiv [XN]_1^+ \longrightarrow [\#]_1^-.$

The M_j object was only generated for mothers who had given birth successfully. The object d_i stores information of the batch, both evolve together to a new object that in subsequent steps will pass to the environment i together with the piglets, LV, where the rules of the piglet rearing process, transition phase and fattening will be carried out.

$$r_{40} \equiv [M_j, d_i]_1^+ \longrightarrow MM_{i,j}[\quad]_1^-, 1 \le i \le bloc, 1 \le j \le q.$$

$$r_{41} \equiv [LV_j, d_i]_1^+ \longrightarrow LLV_{i,j}[\quad]_1^-, 1 \le i \le bloc, 1 \le j \le q.$$

To finish a cycle, the reproduction process of all the mothers belonging to all the batches must be executed. When the reproduction process for the batch i ends, the batch $i + 1$ must be started, until the last batch is reached, at that moment there is a cycle change and the process for the batch 1 starts again.

$$r_{42} \equiv [D_i, cycle_j]_1^+ \longrightarrow D'_{i+1}, cyc_{i,j}[cycle_j]_1^-, 1 \le i < bloc, 1 \le j \le ciclo.$$

$$r_{43} \equiv [D_{bloc}, cycle_j]_1^+ \longrightarrow D'_1, cyc_{bloc,j}[cycle_{j+1}]_1^-, 1 \le j < ciclo.$$

When the last block of the last cycle to simulate is finished, as many Ed objects as batch are generated, these objects will indicate that the processes of the lactation phase can be started.

$r_{44} \equiv [D_{bloc}, cycle_{ciclo}]_1^+ \longrightarrow cyc_{bloc,ciclo}, Ed^{bloc}[\quad]_1^-.$

The objects that have been generated in the different steps and that have not evolved are eliminated so that they do not interfere in the subsequent operation.

$r_{45} \equiv [S_i]_1^+ \longrightarrow [\#]_1^-, 1 \leq i \leq 100.$

$r_{46} \equiv [S']_1^+ \longrightarrow [\#]_1^-.$

Counter evolution

$r_{47} \equiv RM_7[\quad]_1^+ \longrightarrow RM_8\,[\quad]_1^-.$

Step 9 (Step 1) Sending objects to the environment associated with the batch.

Objects associated with the mothers and piglets leave the environment e_{100}, they will move to the environment e_{10i} which is where the rules associated with the rest of the phases of the breeding and fattening process will be executed.

$r_{48} \equiv \left[MM_{i,j}\right]_0^0 \longrightarrow MM_{i,j}[\quad]_0^0, 1 \leq i \leq bloc, 1 \leq j \leq q.$

$r_{49} \equiv \left[LLV_{i,j}\right]_0^0 \longrightarrow LLV_{i,j}[\quad]_0^0, 1 \leq i \leq bloc, 1 \leq j \leq q.$

$r_{50} \equiv \left[cyc_{i,j}\right]_0^0 \longrightarrow cyc_{i,j}[\quad]_0^0, 1 \leq i \leq bloc, 1 \leq j \leq ciclo.$

Elimination of leftover objects.

$r_{51} \equiv [d_i]_1^- \longrightarrow [\#]_1^0, 1 \leq i \leq bloc.$

$r_{52} \equiv \left[M'_j\right]_1^- \longrightarrow M'_j\,[\quad]_1^0, 1 \leq j \leq q.$

The Ed objects that indicate the end of the birth phase must send the information to the different environments where the piglets and mothers are located so that in parallel they initiate the following phases. To ensure that the information will reach all environments, the objects evolve together Bc_i, Ed.

$r_{53} \equiv [Bc_i, Ed]_0^0 \longrightarrow End_i\,[Bc_i]_0^0, 1 \leq i \leq bloc.$

Counter evolution

$r_{54} \equiv RM_8[\quad]_1^- \longrightarrow RM_9\,[\quad]_1^0.$

<u>*Step 10 (Step 2) Sending objects to the environment associated with the batc.*</u>

The index i of the objects is associated to the batch to which it belongs, at this moment all the objects associated with mothers and piglets are going to move from the environment 0 to the environment associated with batch i..

$r_{e1} \equiv \left(MM_{i,j}\right)_{100}(\quad)_{i+100} \longrightarrow (\quad)_{100}\left(ML_j\right)_{i+100}, 1 \leq i \leq bloc, 1 \leq j \leq q.$

$r_{e2} \equiv \left(LLV_{i,j}\right)_{100}(\quad)_{i+100} \longrightarrow (\quad)_{100}\left(LV_j\right)_{i+100}, 1 \leq i \leq bloc, 1 \leq j \leq q.$

$r_{e3} \equiv \left(cyc_{i,j}\right)_{100}(\quad)_{i+100} \longrightarrow (\quad)_{100}\left(cycout_j\right)_{i+100}, 1 \leq i \leq bloc, 1 \leq j \leq ciclo.$

$r_{e4} \equiv \left(End_i\right)_{100}(\quad)_{i+100} \longrightarrow (\quad)_{100}(Ed)_{i+100}, 1 \leq i \leq bloc.$

The object dd_i that keeps information the batch will go to the environment moving to the environment , like the rest of objects, the purpose of this object will be to change the polarity of the membrane 1 of the new environment, to be able to delete the objects whose mission has already ended.
The counter allows the object dd_i to be moved from membrane 1 to the skin membrane.

$r_{55} \equiv [RM_9, dd_i\,]_0^0 \longrightarrow dd_i\,[RM_{10}]_0^0, 1 \leq i \leq bloc.$

<u>*Step 11 (Step 3) Sending objects to the environment associated with the batch.*</u>

Mothers and piglets enter the skin membrane of the new environment.

$r_{56} \equiv LV_j[\,]_0^0 \longrightarrow \left[LV_j\right]_0^0, 1 \leq j \leq q.$

Mothers generate 20 objects of type $IL_{j,1}$ or $IL_{j,0}$, subscript 1 indicates that the mother is infected, while 0 is healthy.

$r_{57} \equiv ML_j[\,]_0^0 \xrightarrow{mi} \left[IL_{j,1}^{20}, MI'_{j,1}\right]_0^0, 1 \leq j \leq q.$

$r_{58} \equiv ML_j[\,]_0^0 \xrightarrow{1-mi} \left[IL_{j,0}^{20}, MI'_{j,0}\right]_0^0, 1 \leq j \leq q.$

In the new environment you will find the objects belonging to mothers and piglets of the different simulated cycles, so you have to keep in the objects the cycle they belonged to, this can be done using the object $cycout_j$.

$$r_{59} \equiv cycout_j[\,]_0^0 \longrightarrow \left[cycout_j^{q\cdot Avl_1^2}\right]_0^0, 1 \leq j \leq ciclo.$$

The Ed object is used to indicate the end of the process and as a counter to synchronize the model.

$$r_{60} \equiv Ed[\,]_0^0 \longrightarrow [\,Ed_1]_0^0.$$

$$r_{e5} \equiv (dd_i)_{100}(\quad)_{i+100} \longrightarrow (\quad)_{100}(dd')_{i+100}, 1 \leq i \leq bloc.$$

Counter evolution

$$r_{61} \equiv [RM_{10} \longrightarrow RM_{11}]_0^0.$$

Step 12 (Step 4) Start of the simulation of the delivery phase for a new batch

$$r_{62} \equiv dd'[\,]_0^0 \longrightarrow [\,dd']_0^0.$$

The counter along with the object D'_i evolve to start a new batch.

$$r_{63} \equiv [RM_{11}, D'_i \longrightarrow RM_0, D_i]_0^0, 1 \leq i \leq bloc.$$

The cycle to which they belong is stored in the objects associated with the piglets and mothers.

$$r_{64} \equiv \left[LV_j, IL_{j,0}, cycout_k \longrightarrow LC_{k,j,0}\right]_0^0, 1 \leq j \leq q, 1 \leq k \leq ciclo.$$

$$r_{65} \equiv \left[LV_j, IL_{j,1}, cycout_k \xrightarrow{li} LC_{k,j,1}\right]_0^0, 1 \leq j \leq q, 1 \leq k \leq ciclo.$$

$$r_{66} \equiv \left[LV_j, IL_{j,1}, cycout_k \xrightarrow{1-li} LC_{k,j,0}\right]_0^0, 1 \leq j \leq q, 1 \leq k \leq ciclo.$$

$$r_{67} \equiv \left[MI'_{j,d}, cycout_k \longrightarrow MIC''_{k,j,d}\right]_0^0, 1 \leq j \leq q, 0 \leq d \leq 1, 1 \leq k \leq ciclo.$$

Step 13 (Step 5) Removing objects

Changing the polarity of the membrane 1.

$$r_{68} \equiv dd'[\,]_1^0 \longrightarrow [\,\#]_1^-.$$

<u>Step 14 (Step 6) Elimination of leftover objects.</u>

$r_{69} \equiv cycout_k[\,]_1^- \longrightarrow [\,\#]_1^0, 1 \leq k \leq ciclo.$

$r_{70} \equiv IL_{j,d}[\,]_1^- \longrightarrow [\,\#]_1^0, 1 \leq j \leq q, 0 \leq d \leq 1.$

Ed object evolution in order to synchronize the model.

$r_{71} \equiv [\,Ed_1 \longrightarrow Ed_2]_0^0.$

$r_{72} \equiv [\,Ed_2 \longrightarrow Ed_3]_0^0.$

$r_{73} \equiv [\,Ed_3 \longrightarrow cycleLR_1]_0^0.$

Phase 2: Lactation

There are two types of management:
Cross fostering that allows to balance the litters weekly once.
Randomization, the pigs are randomly divided between the mothers that have given birth the first week and the two remaining ones can be balanced the litters (cross fostering)

Step 1

When the object $cycleLR_j$ simultaneously enters the skin membrane of the environments $e_{100+i}, 1 \leq i \leq 20$, it indicates that it will start in parallel (in all batchs) the simulation of the lactation process corresponding to cycle j. Objects bbx_j are generated that will allow selecting the objects associated with the mothers and piglets to start the process. RC_0 is a counter that will allow synchronizing the model. The objects $cycleLR_j$ save the cycle that is simulating the lactation phase.

$$r_1 \equiv cycleLR_i\ [\ ]_1^0 \longrightarrow\ bbx_1^{(Avl_1+4)\cdot q}[\ RC_0, cycleLR_i\ ]_1^+, 1 \leq i \leq ciclo.$$

Step 2

In the case of randomized, $gm = 1$, the objects associated with the piglets, LC, enter the membrane 1, otherwise cross fostering evolve to objects of type L in the skin membrane itself.

$$r_2 \equiv LC_{i,j,d}, bbx_i\ [\ ]_1^+ \xrightarrow{gm} \left[LC_{i,j,d}\ \right]_1^0, 1 \leq j \leq q, 0 \leq d \leq 1, 1 \leq i \leq ciclo.$$

$$r_3 \equiv LC_{i,j,d}, bbx_i\ [\ ]_1^+ \xrightarrow{1-gm} L_{i,j,d}\ [\ ]_1^0, 1 \leq j \leq q, 0 \leq d \leq 1, 1 \leq i \leq ciclo.$$

Objects associated with mothers generate new objects in the case of random management.

$$r_4 \equiv MIC''_{i,j,d}, bbx_i\ [\ ]_1^+ \xrightarrow{gm} MI''_{i,j,d}\left[am_j, a_j^{Avl_1-2}\right]_1^0, 1 \leq j \leq q, 0 \leq d \leq 1, 1 \leq i \leq ciclo.$$

$$r_5 \equiv MIC''_{i,j,d}, bbx_i\ [\ ]_1^+ \xrightarrow{1-gm} MI''_{i,j,d}\ [\quad]_1^0, 1 \leq j \leq q, 0 \leq d \leq 1, 1 \leq i \leq ciclo.$$

Evolution counter

$r_6 \equiv \ [RC_0\ ]_1^+ \ \longrightarrow [RC_1]_1^0.$

<u>*Setp 3 to 7*</u>

Piglets are assigned randomly to mothers who have given birth.

$r_7 \equiv \ \left[LC_{i,jj,d}, a_j\ \right]_1^0 \xrightarrow{gm} L_{i,j,d}[\quad]_1^0, 1 \leq j \leq q, 1 \leq jj \leq q, 0 \leq d \leq 1, 1 \leq i \leq ciclo.$

$r_8 \equiv \ \left[LC_{i,jj,d}, am_j\ \right]_1^0 \xrightarrow{gm} L_{i,j,d}\left[am_j, a_j\right]_1^0, 1 \leq j \leq q, 1 \leq jj \leq q, 0 \leq d \leq 1, 1 \leq i \leq ciclo.$

$r_9 \equiv \ [RC_i \ \longrightarrow RC_{i+1}]_1^0, 1 \leq i \leq 4.$

$r_{10} \equiv \ [RC_5\ ]_1^0 \longrightarrow [RC_6]_1^-.$

<u>*Step 8*</u>

In the case of choosing randomization in this step it has already finished, so the surplus objects that have been created for this purpose must be eliminated and the cross fostering started.

$r_{11} \equiv \ [RC_6, cycleLR_i\ ]_1^- \longrightarrow cycleLCF_i[\quad]_1^0, 1 \leq i \leq ciclo.$

$r_{12} \equiv \ \left[am_{\,j}\right]_1^- \xrightarrow{gm} [\#]_1^0, 1 \leq j \leq q.$

$r_{13} \equiv \ \left[a_{\,j}\right]_1^- \xrightarrow{gm} [\#]_1^0, 1 \leq j \leq q.$

$r_{14} \equiv \ bbx_{\,i}[\quad]_1^- \longrightarrow [\#]_1^0, 1 \leq i \leq ciclo.$

<u>*Step 9*</u>

The necessary objects are generated to start the cross fostering

$r_{15} \equiv \ cycleLCF_{\,i}[\quad]_1^0 \longrightarrow RF_0, bx_i^{(Avl_1+2)\cdot q}\left[AV_1^q, CF_1, cycleLL_i\right]_1^+, 1 \leq i \leq ciclo.$

<u>*Step de 10 to 15*</u>

Enter the objects associated with the piglets in the membrane 1, the first index that kept the information of the cycle now goes to save the number of times that has changed mother, generates an object of type B that will allow to count the number of piglets what each mother has.

$r_{16} \equiv L_{j,i,d}, bx_j\, [\,]_1^+ \longrightarrow \left[L_{0,i,d}, B_{i,1}\ \right]_1^0, 1 \leq i \leq q, 0 \leq d \leq 1, 1 \leq j \leq ciclo.$

The objects associated with the mothers evolve losing the index associated with the cycle.

$r_{17} \equiv MI''_{i,j,d}, bx_i\, [\,]_1^+ \longrightarrow \left[MI_{j,d}\right]_1^0, 1 \leq j \leq q, 0 \leq d \leq 1, 1 \leq i \leq ciclo.$

If the mother is sick, MD objects are generated that will allow the infection of new individuals.

$r_{18} \equiv MI_{j,d}[\,]_{11}^0 \longrightarrow \left[MI_{j,d}, MD_j^{d\cdot 7\cdot KL}, MD_j'^{7\cdot(1-d)}\ \right]_{11}^0, 1 \leq j \leq q, 0 \leq d \leq 1.$

Evolution of the counter.

$r_5 \equiv [RF_i \longrightarrow RF_{i+1}]_0^0, 0 \leq i \leq 5.$

The number of piglets per mother is counted.

$r_{20} \equiv \left[B_{i,j}, B_{i,k} \longrightarrow B_{i,j+k}\right]_0^0, 0 \leq i \leq q, 1 \leq j \leq 8, 1 \leq k \leq 16.$

<u>*Step 16*</u>

The counter used for the specific part of RF fostering, evolves to the R counter that will be used in the common part to the two types of management.

$r_{21} \equiv RF_6[\quad]_1^0 \longrightarrow [\,R_1]_1^+.$

<u>*Step 17*</u>

If the number of piglets that a mother has is higher than the average value, she releases the rest, BL_i, which can be adopted by other mothers.

$r_{22} \equiv \left[B_{i,k}, AV_w \longrightarrow BL_i^{k-floor(Avl_w)}\right]_1^+, floor(Avl_w) \leq k \leq 20, 1 \leq i \leq q, 1 \leq w \leq 3.$

On the opposite, if a mother does not reach the minimum value, she generates as many objects of the type BN_i as piglets she lacks to reach the value.

$$r_{23} \equiv \left[B_{i,k}, AV_w \longrightarrow BN_i^{k-floor(Avl_w-k)}\right]_1^+, 1 \leq k < floor(Avl_w), 1 \leq i \leq q, 1 \leq w \leq 3.$$

Evolution of the counter.

$$r_{24} \equiv [R_1 \longrightarrow R_2]_1^+.$$

Step 18

Objects belonging to the surplus piglets, F , are randomly selected and the information is stored in the give object, in case it is the case that the piglet is not adopted, it is returned to the mother from whom it has emerged.

$$r_{25} \equiv \left[BL_i, L_{r,i,k} \longrightarrow give_{i,k}, F_{r,k}\right]_1^+, 1 \leq i < q, 0 \leq r \leq 2, 0 \leq k \leq 1.$$

$$r_{26} \equiv [R_2 \longrightarrow R_3]_1^+.$$

Step 19

Free piglets are assigned to mothers who did not reach the average value.

$$r_{27} \equiv \left[BN_i, F_{r,d}\right]_1^+ \longrightarrow \left[L_{r+1,i,d}\right]_1^-, 1 \leq i < q, 0 \leq r \leq 2, 0 \leq d \leq 1.$$

Weekly die a number of piglets, this process is carried out using the object O that is generated in this step.

$$r_{28} \equiv [R_3, CF_i]_1^+ \longrightarrow \left[R_4, O_i^{q\cdot(Avl_i+2)}, CF_i\right]_1^-, 1 \leq i < 3.$$

Step 20

Elimination of objects left over if there were not enough piglets to be adopted.

$$r_{29} \equiv [BN_i]_1^- \longrightarrow [\#]_1^0, 1 \leq i < q.$$

Simulation of the process of mortality and return of piglets to mothers of origin, in case they have not been adopted.

$$r_{30} \equiv L_{i,j,d}, O_k \left[\quad \right]_{11}^{0} \xrightarrow{ml_k + d \cdot LL \cdot (1 - mld_k)} [\#]_{11}^{0}, 0 \le i \le k, 1 \le j < q, 1 \le k \le 3, 0 \le d \le 1.$$

$$r_{31} \equiv L_{i,j,d}, O_k \left[\quad \right]_{11}^{0} \xrightarrow{1 - ml_k + d \cdot LL \cdot (1 - mld_k)} B_{j,1} \left[L_{i,j,d}, MD_j^{7 \cdot KL \cdot d}, MD'^{7 \cdot (1-d)}_j \right]_{11}^{0}, 0 \le i \le k, 1 \le j < q, 1 \le k \le 3, 0 \le d \le 1.$$

$$r_{32} \equiv F_{r,d}, give_{i,d}, O_k \left[\quad \right]_{11}^{0} \xrightarrow{ml_k + d \cdot LL \cdot (1 - mld_k)} [\#]_{11}^{0}, 0 \le r \le k, 1 \le i < q, 1 \le k \le 3, 0 \le d \le 1.$$

$$r_{33} \equiv F_{r,d}, give_{i,d}, O_k \left[\quad \right]_{11}^{0} \xrightarrow{1 - ml_k + d \cdot LL \cdot (1 - mld_k)} B_{i,1} \left[L_{r+1,i,d}, MD_j^{7 \cdot KL \cdot d}, MD'^{7 \cdot (1-d)}_j \right]_{11}^{0}, 0 \le r \le k, 1 \le i < q, 1 \le k \le 3, 0 \le d \le 1.$$

Elimination of objects that are left over and evolution of the counter.

$r_{34} \equiv [AV_w]_1^- \rightarrow [\#]_1^0, 1 \le w \le 3.$

$r_{35} \equiv bx_i[\quad]_1^- \rightarrow [\#]_1^0, 1 \le i \le 3.$

$r_{36} \equiv [R_4]_1^- \rightarrow [R_5]_1^0.$

Step 21

Elimination of leftover objects.

$r_{37} \equiv \left[give_{j,k} \rightarrow \# \right]_1^0, 1 \le j \le q, 0 \le k \le 1.$

$r_{38} \equiv [O_k \rightarrow \#]_1^0, 1 \le k \le 3.$

Infection of piglets infected by sick mothers.

$$r_{39} \equiv \left[L_{i,j,0}, MD_j^n, MD'^{7-n}_j \right]_{11}^{0} \xrightarrow{(1-pL)^n} \left[L_{i,j,0} \right]_{11}^{+}, 0 \le i \le 3, 1 \le j \le q, 0 \le n \le 7.$$

$$r_{40} \equiv \left[L_{i,j,0}, MD_j^n, MD'^{7-n}_j \right]_{11}^{0} \xrightarrow{1-(1-pL)^n} \left[L_{i,j,1} \right]_{11}^{+}, 0 \le i \le 3, 1 \le j \le q, 0 \le n \le 7.$$

$$r_{41} \equiv \left[L_{i,j,1}, MD_j^n, MD'^{7-n}_j \right]_{11}^{0} \longrightarrow \left[L_{i,j,1} \right]_{11}^{+}, 0 \le i \le 3, 1 \le j \le q, 0 \le n \le 7.$$

$r_{42} \equiv R_5[\quad]_{11}^0 \rightarrow R_6[\quad]_{11}^+.$

Step 22 to 26

In order to synchronize, having the same number of steps, the two types of management, only the meter evolves.

$r_{43} \equiv [R_i \longrightarrow R_{i+1}]_1^0, 6 \leq i \leq 9.$

Elimination of leftover infectious objects.

$r_{44} \equiv \left[MD_j \longrightarrow \#\right]_1^0, 1 \leq j \leq q.$

$r_{45} \equiv \left[MD'_j \longrightarrow \#\right]_1^0, 1 \leq j \leq q.$

Step 27

Up to this step all the processes belonging to the first week have been executed, now the ones of the following week must be started, if another cros fostering is carried out the object CF_1 is generated, otherwise the object CF_0.

$r_{46} \equiv$
$R_{10}, CF_i[\quad]_{11}^{+} \rightarrow R_{11}, CF_i, FC_1^{q \cdot WeekCross_{i+1}}, FC_0^{q \cdot (1-WeekCross_{i+1})}\left[clean^{q \cdot (Avl_1+4)}\right]_{11}^{+}, 1 \leq i \leq 2.$

When the end of the lactation period is reached, FC objects are not generated. The one generated is the object FCC.

$r_{47} \equiv R_{10}, CF_3[\quad]_{11}^{+} \rightarrow R_{11}, CF_3, FCC_0^{q}\left[clean^{q \cdot (Avl_1+4)}\right]_{11}^{+}.$

Step 28

They leave the objects associated with piglets and mothers of the deepest membrane.

$r_{48} \equiv \left[L_{i,j,d}, clean\right]_{11}^{+} \rightarrow LW_{i,j,d}[\quad]_{11}^{-}, 0 \leq i \leq 3, 1 \leq j \leq q, 0 \leq d \leq 1.$

$r_{49} \equiv \left[MI_{j,d}, clean\right]_{11}^{+} \rightarrow MII_{j,d}[\quad]_{11}^{-}, 1 \leq j \leq q, 0 \leq d \leq 1.$

Evolution in the case of not having reached the end of the lactation phase.

$r_{50} \equiv \left[FC_k, B_{j,b}\right]_1^{+} \rightarrow \left[B_{j,b}^{k}\right]_1^{-}, 1 \leq j \leq q, 0 \leq k \leq 1, 1 \leq b \leq 20.$

Evolution in the case of having reached the end of the lactation phase.

$r_{51} \equiv \left[FCC_0, B_{j,b}\right]_1^+ \rightarrow [\#]_1^-, 1 \leq j \leq q, 1 \leq b \leq 20$.

$r_{52} \equiv [R_{11}]_1^+ \rightarrow [R_{12}]_1^-$.

<u>*Step 29*</u>

$r_{53} \equiv [clean]_{11}^- \rightarrow [\#]_{11}^0$.

In the case of not having cross fostering the following week the counter $C'$is generated temporarily, otherwise it is followed by the counter R that is reinitialized.

$r_{54} \equiv [R_{12}, CF_i]_1^- \rightarrow \left[CF_{i+1}, {R'}_0^{WeekCross_{i+1}}, {C'}_0^{1-WeekCross_{i+1}}, AV_{i+1}^q\right]_1^0, 1 \leq i \leq 2$.

Evolution in the case of end of a cycle and start of the next.

$r_{55} \equiv \left[R_{12}, CF_3, cycleLL_j\right]_1^- \rightarrow cycleL'_{j+1}\left[cyc_j^{q\cdot(Avl_1+2)}\right]_1^+, 1 \leq j \leq ciclo - 1$.

Evolution in the case of the end of the last cycle.

$r_{56} \equiv [R_{12}, CF_3, cycleLL_{ciclo}]_1^- \rightarrow PAS_1\left[cyc_{ciclo}^{q\cdot(Avl_1+2)}\right]_1^+$.

Removal of objects

$r_{57} \equiv [FCC_0]_1^- \rightarrow [\#]_1^+, 0 \leq k \leq 1$.

$r_{58} \equiv [FC_k]_1^- \rightarrow [\#]_1^0, 0 \leq k \leq 1$.

<u>*Step 30*</u>

Evolution of objects to start the cross-fostering process again.

$r_{59} \equiv [cycleL'_i \rightarrow cycleL''_i]_0^0, 2 \leq i \leq 3$.

$r_{60} \equiv \left[LW_{i,j,d} \rightarrow L_{i,j,d}\right]_1^0, 0 \leq i \leq 3, 1 \leq j \leq q, 0 \leq d \leq 1$.

$r_{61} \equiv \left[MII_{j,d} \rightarrow MI_{j,d}\right]_1^0, 1 \leq j \leq q, 0 \leq d \leq 1$.

$r_{62} \equiv [R'_0 \rightarrow R_0]_1^0$.

$r_{63} \equiv [C'_0 \rightarrow C_0]_1^0$.

Step 31

$r_{64} \equiv [cycleL''_i \rightarrow cycleLR_i]_0^0$, $2 \leq i \leq 3$.

$r_{65} \equiv [R_0]_1^0 \rightarrow [R_1]_1^+$.

$r_{66} \equiv [C_i \rightarrow C_{i+1}]_1^0, 0 \leq i \leq 2$.

Moment in which the processes are synchronized in the case of having or not done cross fostering a week.

$r_{67} \equiv [C_3, CF_i]_1^0 \rightarrow \left[R_4, O_i^S, CF_i\right]_1^-$, $1 \leq i \leq 3$.

Rules that are carried out at the end of the cycle, when the piglets reach 21 days of life and must start the transition phase. Each piglet is associated with an object of type XT that will be the one that evolved, LL objects are also generated that keep information respectively of the cycle, movements, disease and mother to which the pig belonged at the end of the piglet cycle. The W objects that store the information of the weight of each animal.

$$r_{68} \equiv \left[LW_{i,j,d}, cyc_c\right]_1^+ \rightarrow XT_{c,j,d}, LL_{c,i,j,d}, W_{c,i,j,d}^{Lweight_{j\cdot(1+d)}-(loss\cdot i+d\cdot lossdiseadeL)}[\quad]_1^0, 0 \leq i \leq 3, 1 \leq j \leq q, 0 \leq d \leq 1, 1 \leq c \leq ciclo.$$

Evolution of the objects associated with the mothers, this information will be transmitted to the transition phase to know the mothers who have completed the lactaction phase.

$r_{69} \equiv \left[MII_{j,d}, cyc_c\right]_1^+ \rightarrow Mt'_{c,j}[\quad]_1^0$,$1 \leq j \leq q, 0 \leq d \leq 1, 1 \leq c \leq ciclo$.

Elimination of objects.

$r_{70} \equiv [cyc_c \rightarrow \#]_1^0$,$1 \leq c \leq ciclo$.

Generation of the object $cycleT$ indicating that the transition phase can already be started.

$r_{71} \equiv [PAS_1 \rightarrow cycleT_1]_0^0$.

Phase 3: Transition phase

As happened in the lactation phase, all the environments receive simultaneously the object $cycleT_c$ that indicates that the pigs that belong to cycle c of the 20 batches will initiate the transition phase.

Step 1

Two object objects are generated RI_0 (counter) and bt_c, the second will allow selecting the pigs and knowing the mothers who had raised them.

$r_1 \equiv cycleT_c[\quad]_2^0 \longrightarrow bt_c^{(Avl_1+10)\cdot 20\cdot q}, RI_0\ [cycleT_c]_2^0, 1 \le c \le ciclo.$

Step 2

It is recorded in the object MTT_j the mothers who had carried out the breastfeeding process.

$r_2 \equiv MT_j, MT'_{c,j}, bt_c[\quad]_4^0 \longrightarrow MTT_j\ [MT_j]_4^+, 1 \le j \le q, 1 \le c \le ciclo.$

$r_3 \equiv\ RI_0[\quad]_2^0 \longrightarrow RI_1\ [\quad]_2^-.$

Step 3

The objects $LL_{c,i,j,d}$ and $W_{c,i,j,d}$ that had been generated to save the corresponding information at the end of the lactation phase are eliminated.

$r_4 \equiv LL_{c,i,j,d}[\quad]_2^- \longrightarrow\ [\#]_2^-, 1 \le c \le ciclo, 0 \le i \le 3, 1 \le j \le q, 0 \le d \le 1.$

$r_5 \equiv W_{c,i,j,d}[\quad]_2^- \longrightarrow\ [\#]_2^-, 1 \le c \le ciclo, 0 \le i \le 3, 1 \le j \le q, 0 \le d \le 1.$

The objects MT_j, associated with mothers who had not performed the breastfeeding process, enter membrane 4, where a copy is kept for all mothers.

$r_6 \equiv MT_j[\quad]_4^+ \longrightarrow\ [MT_j]_4^+, 1 \le j \le q.$

Management in the transition phase can be done in two ways: grouping the piglets by mothers or randomly, in both cases they will be separated by sex.

Case 1 Distribution of pens by mothers

Step 4 to 11

A pen is associated to a mother, objects $PenM_{i,j}$ are generated that will be used later so that the pigs of the mother j go to pen i.

$$r_7 \equiv MTT_j, PN_i[\quad]_2^- \xrightarrow{gt_1} PN_i\left[PenM_{i,j}^{20}\right]_2^-, 1 \leq j \leq q, 1 \leq i \leq floor\left(\frac{Tpen}{2}\right).$$

Counter evolution

$$r_8 \equiv \left[RI_i \xrightarrow{gt_1} RI_{i+1}\right]_0^0, 1 \leq i \leq 8.$$

Step 12

Evolution of the counter to indicate that each pig can now be assigned its pen, each mother has been assigned a pen number that is where her piglets will go.

$$r_9 \equiv RI_9[\quad]_2^- \xrightarrow{gt_1} RI_{10}[\quad]_2^+.$$

Step 13

Enter the objects assigned to the pigs of the cycle that is simulating in the membrane 1 to assign it in the next step the pen.

$$r_{10} \equiv bt_c, XT_{c,j,d}[\quad]_2^+ \xrightarrow{gt_1} \left[XT'_{j,d}\right]_2^+, 1 \leq c \leq ciclo, 1 \leq j \leq q, 0 \leq d \leq 1.$$

$$r_{11} \equiv \left[RI_{10} \xrightarrow{gt_1} RI_{11}\right]_0^0.$$

Step 14

A pen is assigned to each pig separating the males from the females.

$$r_{12} \equiv \left[PenM_{i,j}, XT'_{j,d} \xrightarrow{0.5 \cdot gt_1} YT_{i,d}, IT'^{d}_{i}, ST_i^{(1-d)}\right]_2^+, 1 \leq j \leq q, 1 \leq i \leq floor\left(\frac{Tpen}{2}\right), 0 \leq d \leq 1.$$

$$r_{13} \equiv \left[PenM_{i,j}, XT'_{j,d} \xrightarrow{0.5 \cdot gt_1} YT_{Tpen+1-i,d}, IT'^{d}_{Tpen+1-i}, ST_{Tpen+1-i}^{(1-d)}\right]_2^+, 1 \leq j \leq q, 1 \leq i \leq floor\left(\frac{Tpen}{2}\right), 0 \leq d \leq 1.$$

Counter evolution

$$r_{14} \equiv \left[RI_{11} \xrightarrow{gt_1} RI_{12}\right]_0^0.$$

Case 2 Random distribution

<u>Step 4</u>

We will generate objects that store the information of each pen so that in a later step these objects will be randomly assigned to the pigs that have finished the lactation phase. Three types of objects are generated, PN', PN'' and PN''', to get a distribution of pigs as similar as possible for each pen.

$$r_{15} \equiv PN_i[\quad]_2^- \xrightarrow{gt_2} \left[PN''^{\frac{q\cdot Avl_1}{Tpen}}_i, PN'_i, PN'''^2_i\right]_2^-, \; 1 \le i \le floor\left(\frac{Tpen}{2}\right).$$

Enter the objects associated with the pigs in the membrane 2, which is where the transition process takes place.

$$r_{16} \equiv bt_c, XT_{c,j,d}[\quad]_2^- \xrightarrow{gt_2} [XT''_d]_2^-, \; 1 \le c \le ciclo, 1 <= j <= q, 0 \le d \le 1.$$

<u>Step 5 to 15</u>

The object $YT_{i,d}$stores information of the pen assigned to each pig in its first index and in its second index if it is infected or not.

$$r_{17} \equiv \left[PN''_i, XT''_d \xrightarrow{0.5\cdot gt_2} YT_{i,d}, IT'^d_i, ST_i^{1-d}\right]_2^-, \; 1 \le i \le floor\left(\frac{Tpen}{2}\right), 0 \le d \le 1.$$

$$r_{18} \equiv \left[PN''_i, XT''_d \xrightarrow{0.5\cdot gt_2} YT_{Tpen+1-i,d}, IT'^d_{Tpen+1-i}, ST^{1-d}_{Tpen+1-i}\right]_2^-, \; 1 \le i \le floor\left(\frac{Tpen}{2}\right), 0 \le d \le 1.$$

$$r_{19} \equiv \left[PN'''_i, XT''_d \xrightarrow{0.5\cdot gt_2} PN'''_i, YT_{i,d}, IT'^d_i, ST_i^{1-d}\right]_2^-, \; 1 \le i \le floor\left(\frac{Tpen}{2}\right), 0 \le d \le 1.$$

$$r_{20} \equiv \left[PN'''_i, XT''_d \xrightarrow{0.5\cdot gt_2} PN'''_i, YT_{Tpen+1-i,d}, IT'^d_{Tpen+1-i}, ST^{1-d}_{Tpen+1-i}\right]_2^-, \; 1 \le i \le floor\left(\frac{Tpen}{2}\right), 0 \le d \le 1.$$

$r_{21} \equiv \left[PN'_i, XT''_d \xrightarrow{0.5\cdot gt_2} PN'_i, YT_{i,d}, IT'^{d}_{i}, ST_i^{1-d}\right]_2^-, \; 1 \leq i \leq floor\left(\frac{Tpen}{2}\right), 0 \leq d \leq 1.$

$r_{22} \equiv \left[PN'_i, XT''_d \xrightarrow{0.5\cdot gt_2} PN'_i, YT_{Tpen+1-i,d}, IT'^{d}_{Tpen+1-i}, ST^{1-d}_{Tpen+1-i}\right]_2^-, \; 1 \leq i \leq floor\left(\frac{Tpen}{2}\right), 0 \leq d \leq 1.$

Evolution counter

$r_{23} \equiv \left[RI_i \xrightarrow{gt_2} RI_{i+1}\right]_0^0, 1 \leq i \leq 10.$

$r_{24} \equiv RI_{11} [\,]_2^- \xrightarrow{gt_2} RI_{12}[\quad]_2^+ .$

Step 16

It sells the objects that have to be used and those that are not going to be used are eliminated.

$r_{25} \equiv [PN'_i\,]_2^+ \xrightarrow{gt_2} PN'_i[\quad]_2^0, 1 \leq i \leq floor\left(\frac{Tpen}{2}\right).$

$r_{26} \equiv [PN''_i\,]_2^+ \xrightarrow{gt_2} [\#]_2^0, 1 \leq i \leq floor\left(\frac{Tpen}{2}\right).$

$r_{27} \equiv [PN'''_i\,]_2^+ \xrightarrow{gt_2} [\#]_2^0, 1 \leq i \leq floor\left(\frac{Tpen}{2}\right).$

$r_{28} \equiv PN'_i[\,]_4^+ \xrightarrow{gt_2} [PN'_i]_4^0, 1 \leq i \leq floor\left(\frac{Tpen}{2}\right).$

Common part to the two types of management

Step 17

In a randomized way a state is assigned to the sick animal, they are infected animals in the previous phase. The disease may be in the incubation phase, in the transmission or recovery phase.

$r_{29} \equiv [IT'_i\,]_2^+ \xrightarrow{\frac{(21-InfT)}{21}} [RT_i]_2^0, 1 \leq i \leq Tpen\,.$

$r_{30} \equiv [IT'_i\,]_2^+ \xrightarrow{\frac{(InfT-IncT)}{21}} \left[IT_{i,avIncfInft}\right]_2^0, 1 \leq i \leq Tpen\,.$

$$r_{31} \equiv [IT'_i\ ]_2^{+} \xrightarrow{\frac{IncT}{21}} \left[IT_{i,avInc}\right]_2^{0}, 1 \leq i \leq Tpen\ .$$

Counter evolution

$$r_{32} \equiv RI_{12}\ [\ ]_2^{+} \longrightarrow RI_{13}[\quad]_2^{0}\ .$$

Step 18

Elimination of unnecessary objects

$$r_{33} \equiv \left[PenM_{i,j}\ \right]_2^{0} \longrightarrow [\#]_2^{-}, 1 \leq j \leq q, 1 \leq i \leq \frac{Tpen}{2}\ .$$

Daily simulation of the disease. Infected animals that do not transmit.

$$r_{34} \equiv \left[IT_{i,t}\ \right]_2^{0} \longrightarrow \left[IT_{i,t+1}\right]_2^{-}, 1 \leq i \leq Tpen, 0 \leq t \leq IncT\ .$$

Infected animals that transmit the disease.

$$r_{35} \equiv \left[IT_{i,t}\ \right]_2^{0} \longrightarrow \left[IT_{i,t+1}, INFG_i^{KT\cdot ctg}\right]_2^{-}, 1 \leq i \leq Tpen, IncT < t < IncT + InfT\ .$$

Animals that have completed the infective period can be recovered or killed.

$$r_{36} \equiv \left[IT_{i,IncT+InfT}\ \right]_2^{0} \xrightarrow{(1-LT)} \left[RT_i, INFG_i^{KT\cdot ctg}\right]_2^{-}, 1 \leq i \leq Tpen\ .$$

$$r_{37} \equiv \left[IT_{i,IncT+InfT}\ \right]_2^{0} \xrightarrow{LT} \left[LT_i, INFG_i^{KT\cdot ctg}\right]_2^{-}, 1 \leq i \leq Tpen.$$

Evolution of the counter.

$$r_{38} \equiv RI_{13}\ [\ ]_2^{0} \longrightarrow RI_{14}[\quad]_2^{-}\ .$$

Step 19

The infected animals that are in a pen can transmit the disease to animals of the pen itself or to nearby pens, the objects INF_i will be used to transmit the disease to pigs that are in the pen .

$$r_{39} \equiv \left[INFG_i \xrightarrow{\frac{2}{7}} INF_i\right]_2^{-}, 1 < i < Tpen, i \neq \frac{Tpen}{2}, i \neq \frac{Tpen}{2} + 1.$$

$$r_{40} \equiv \left[INFG_i \xrightarrow{\frac{1}{7}} INF_{i-1}\right]_2^{-}, 1 < i < Tpen, i \neq \frac{Tpen}{2}, i \neq \frac{Tpen}{2} + 1.$$

$$r_{41} \equiv \left[INFG_i \xrightarrow{\frac{1}{7}} INF_{i+1} \right]_2^- ,1 < i < Tpen, i \neq \frac{Tpen}{2}, i \neq \frac{Tpen}{2} + 1.$$

$$r_{42} \equiv \left[INFG_i \xrightarrow{\frac{1}{7}} INF_{Tpen-i+1} \right]_2^- ,1 < i < Tpen, i \neq \frac{Tpen}{2}, i \neq \frac{Tpen}{2} + 1.$$

$$r_{43} \equiv \left[INFG_i \xrightarrow{\frac{1}{7}} INF_{Tpen-i+2} \right]_2^- ,1 < i < Tpen, i \neq \frac{Tpen}{2}, i \neq \frac{Tpen}{2} + 1.$$

$$r_{44} \equiv \left[INFG_i \xrightarrow{\frac{1}{7}} INF_{Tpen-i} \right]_2^- ,1 < i < Tpen, i \neq \frac{Tpen}{2}, i \neq \frac{Tpen}{2} + 1.$$

$$r_{45} \equiv \left[INFG_1 \xrightarrow{\frac{2}{5}} INF_1 \right]_2^- .$$

$$r_{46} \equiv \left[INFG_1 \xrightarrow{\frac{1}{5}} INF_2 \right]_2^- .$$

$$r_{47} \equiv \left[INFG_1 \xrightarrow{\frac{1}{5}} INF_{Tpen} \right]_2^- .$$

$$r_{48} \equiv \left[INFG_1 \xrightarrow{\frac{1}{5}} INF_{Tpen-1} \right]_2^- .$$

$$r_{49} \equiv \left[INFG_{\frac{Tpen}{2}} \xrightarrow{\frac{2}{5}} INF_{\frac{Tpen}{2}} \right]_2^- .$$

$$r_{50} \equiv \left[INFG_{\frac{Tpen}{2}} \xrightarrow{\frac{1}{5}} INF_{\frac{Tpen}{2}-1} \right]_2^- .$$

$$r_{51} \equiv \left[INFG_{\frac{Tpen}{2}} \xrightarrow{\frac{1}{5}} INF_{\frac{Tpen}{2}+1} \right]_2^- .$$

$$r_{52} \equiv \left[INFG_{\frac{Tpen}{2}} \xrightarrow{\frac{1}{5}} INF_{\frac{Tpen}{2}+2} \right]_2^- .$$

$$r_{53} \equiv \left[INFG_{\frac{Tpen}{2}+1} \xrightarrow{\frac{2}{5}} INF_{\frac{Tpen}{2}+1} \right]_2^- .$$

$$r_{54} \equiv \left[INFG_{\frac{Tpen}{2}+1} \xrightarrow{\frac{1}{5}} INF_{\frac{Tpen}{2}-1} \right]_2^- .$$

$$r_{55} \equiv \left[INFG_{\frac{Tpen}{2}+1} \xrightarrow{\frac{1}{5}} INF_{\frac{Tpen}{2}} \right]_2^- .$$

$$r_{56} \equiv \left[INFG_{\frac{Tpen}{2}+1} \xrightarrow{\frac{1}{5}} INF_{\frac{Tpen}{2}+2} \right]_2^- .$$

$$r_{57} \equiv \left[INFG_{Tpen} \xrightarrow{\frac{2}{5}} INF_{Tpen} \right]_2^- .$$

$$r_{58} \equiv \left[INFG_{Tpen} \xrightarrow{\frac{1}{5}} INF_{1} \right]_2^- .$$

$$r_{59} \equiv \left[INFG_{Tpen} \xrightarrow{\frac{1}{5}} INF_{2} \right]_2^- .$$

$$r_{60} \equiv \left[INFG_{Tpen} \xrightarrow{\frac{1}{5}} INF_{Tpen-1} \right]_2^- .$$

Counter evolution An object, day, is generated that records the days that have passed in the transition phase

$$r_{61} \equiv [RI_{14} \longrightarrow RI_{15}, day]_0^0.$$

Step 20

An infected object in contact with an already infected animal has no effect.

$$r_{62} \equiv IT_{i,t}, INF_i[\quad]_{22}^0 \longrightarrow \left[IT_{i,t}\right]_{22}^0, 1 \leq i \leq Tpen, 0 \leq t \leq IncT + infT.$$

If the infectious object is in contact with a recovered animal, it has no effect

$$r_{63} \equiv RT_i, INF_i[\quad]_{22}^0 \longrightarrow [RT_i]_{22}^0, 1 \leq i \leq Tpen.$$

Si el objeto infeccioso esta e contacto con un animal susceptible puede o no infectarlo.

$$r_{64} \equiv ST_i, INF_i[\quad]_{22}^0 \xrightarrow{pT} \left[IT_{i,0}\right]_{22}^0, 1 \leq i \leq Tpen.$$

$$r_{65} \equiv ST_i, INF_i[\quad]_{22}^0 \xrightarrow{(1-pT)} [ST_i]_{22}^0, 1 \leq i \leq Tpen.$$

When we have 42 objects of the day type, it indicates that the phase for the simulated cycle has ended.

$$r_{66} \equiv [day^{42} \longrightarrow end]_0^0.$$

Evolution of the counter.

$r_{67} \equiv\ [RI_{15} \longrightarrow RI_{16}]_0^0.$

Step 21

Evolution of the objects related to the disease associated with each pig.

$r_{68} \equiv\ [IT_{i,t}]_{22}^0 \longrightarrow ITT_{i,t}[\quad]_{22}^0, 1 \leq i \leq Tpen, 0 \leq t \leq IncT + InfT.$

$r_{69} \equiv\ [RT_i]_{22}^0 \longrightarrow RTT_i[\quad]_{22}^0, 1 \leq i \leq Tpen.$

$r_{70} \equiv\ [ST_i]_{22}^0 \longrightarrow STT_i[\quad]_{22}^0, 1 \leq i \leq Tpen.$

$r_{71} \equiv\ RI_{16}, end[\quad]_2^- \longrightarrow RI_{17}[end]_2^+.$

$r_{72} \equiv\ RI_{16}, day[\quad]_2^- \longrightarrow RI_{13}, day[\quad]_2^+.$

Step 22

Elimination of leftover objects and evolution of those who must continue with the process.

$r_{73} \equiv\ [INF_i]_2^0 \longrightarrow [\#]_2^-, 1 \leq i \leq Tpen.$

$r_{74} \equiv\ [INF_i]_2^+ \longrightarrow [\#]_2^+, 1 \leq i \leq Tpen.$

$r_{75} \equiv\ [ITT_{i,t}]_2^0 \longrightarrow [IT_{i,t+1}]_2^-, 1 \leq i \leq Tpen, 0 \leq t \leq IncT.$

$r_{76} \equiv\ [ITT_{i,t}]_2^0 \longrightarrow [IT_{i,t+1}, INFG_i^{KT\cdot ctg}]_2^-, 1 \leq i \leq Tpen, IncT < t < IncT + infT.$

$r_{77} \equiv\ [ITT_{i,IncT+InfT}]_2^0 \xrightarrow{1-LT} [RT_i, INFG_i^{KT\cdot ctg}]_2^-, 1 \leq i \leq Tpen.$

$r_{78} \equiv\ [ITT_{i,IncT+InfT}]_2^0 \xrightarrow{LT} [LT_i, INFG_i^{KT\cdot ctg}]_2^-, 1 \leq i \leq Tpen.$

$r_{79} \equiv\ [IT_{i,t}]_2^0 \longrightarrow [IT_{i,t+1}]_2^-, 1 \leq i \leq Tpen, 0 \leq t \leq IncT.$

$r_{80} \equiv\ [IT_{i,t}]_2^0 \longrightarrow [IT_{i,t+1}, INFG_i^{KT\cdot ctg}]_2^-, 1 \leq i \leq Tpen, IncT < t < IncT + infT.$

$r_{81} \equiv\ [IT_{i,IncT+InfT}]_2^0 \xrightarrow{1-LT} [RT_i, INFG_i^{KT\cdot ctg}]_2^-, 1 \leq i \leq Tpen.$

$r_{82} \equiv\ [IT_{i,IncT+InfT}]_2^0 \xrightarrow{LT} [LT_i, INFG_i^{KT\cdot ctg}]_2^-, 1 \leq i \leq Tpen.$

$r_{83} \equiv\ [STT_i]_2^0 \longrightarrow [ST_i]_2^-, 1 \leq i \leq Tpen.$

$r_{84} \equiv\ [RTT_i]_2^0 \longrightarrow [RT_i]_2^-, 1 \leq i \leq Tpen.$

$r_{85} \equiv\ \left[ITT_{i,t} \longrightarrow IT_{i,t}\right]_2^+, 1 \leq i \leq Tpen, 0 \leq t \leq IncT + infT + 1.$

$r_{86} \equiv\ [RTT_i \longrightarrow RT_i]_2^+, 1 \leq i \leq Tpen.$

$r_{87} \equiv\ [STT_i \longrightarrow ST_i]_2^+, 1 \leq i \leq Tpen.$

$r_{88} \equiv\ RI_{17}[\quad]_2^+ \longrightarrow [RI_{18}]_2^+.$

$r_{89} \equiv\ end[\quad]_{22}^0 \longrightarrow [\#]_2^+.$

<u>Step 23</u>

It is associated with the objects associated with the animals $YT_{i,1}$, the objects associated with the health status of each one of them. First it is done for animals that are not sick.

$r_{90} \equiv\ YT_{i,1}, RT_i[\quad]_{22}^+ \xrightarrow{1-mt} \left[ZT_{i,1,0}\right]_{22}^-, 1 \leq i \leq Tpen.$

$r_{91} \equiv\ YT_{i,0}, ST_i[\quad]_{22}^+ \xrightarrow{1-mt} \left[ZT_{i,0,0}\right]_{22}^-, 1 \leq i \leq Tpen.$

$r_{92} \equiv\ YT_{i,1}, RT_i[\quad]_{22}^+ \xrightarrow{mt} [\#]_{22}^-, 1 \leq i \leq Tpen.$

$r_{93} \equiv\ YT_{i,0}, ST_i[\quad]_{22}^+ \xrightarrow{mt} [\#]_{22}^-, 1 \leq i \leq Tpen.$

$r_{94} \equiv\ YT_{i,1}, LT_i[\quad]_{22}^+ \longrightarrow [\#]_{22}^-, 1 \leq i \leq Tpen.$

$r_{95} \equiv\ RI_{18}[\quad]_{22}^+ \longrightarrow RI_{19}[\quad]_{22}^-.$

<u>Step 24</u>

It is associated with the objects associated with the animals $YT_{i,1}$, the objects associated with the health status of each one of them. Now it is done for sick animals.

$r_{96} \equiv\ YT_{i,1}, IT_{i,t}[\quad]_{22}^- \xrightarrow{1-mt} \left[ZT_{i,1,t}\right]_{22}^0, 1 \leq i \leq Tpen, 0 \leq t \leq IncT + InfT.$

$r_{97} \equiv\ YT_{i,0}, IT_{i,t}[\quad]_{22}^- \xrightarrow{1-mt} \left[ZT_{i,1,t}\right]_{22}^0, 1 \leq i \leq Tpen, 0 \leq t \leq IncT + InfT.$

$r_{98} \equiv\ YT_{i,0}, RT_i[\quad]_{22}^- \xrightarrow{1-mt} \left[ZT_{i,1,0}\right]_{22}^0, 1 \leq i \leq Tpen.$

$r_{99} \equiv\ YT_{i,0}, LT_i[\quad]_{22}^- \longrightarrow [\#]_{22}^0, 1 \leq i \leq Tpen.$

$r_{100} \equiv \ YT_{i,1}, IT_{i,t}[\quad]_{22}^{-} \xrightarrow{mt} [\#]_{22}^{0}, 1 \leq i \leq Tpen, 0 \leq t \leq IncT + InfT.$

$r_{101} \equiv \ YT_{i,0}, IT_{i,t}[\quad]_{22}^{-} \xrightarrow{mt} [\#]_{22}^{0}, 1 \leq i \leq Tpen, 0 \leq t \leq IncT + InfT.$

$r_{102} \equiv \ YT_{i,0}, RT_{i}[\quad]_{22}^{-} \xrightarrow{mt} [\#]_{22}^{0}, 1 \leq i \leq Tpen.$

$r_{103} \equiv \ RI_{19}[\quad]_{22}^{-} \longrightarrow RI_{20}[\quad]_{22}^{0}.$

Step 25

The objects must leave the inner membrane, they have to reach the skin membrane that is where they will start the final fattening process.

$r_{104} \equiv \ \left[ZT_{i,j,t}\right]_{22}^{0} \longrightarrow ZT_{i,j,t}[\quad]_{22}^{0}, 1 \leq i \leq Tpen, 0 \leq j \leq 1{,}0 \leq t \leq IncT + InfT.$

$r_{105} \equiv \ \left[RI_{20}, cycleT_{c} \longrightarrow btt_{c}^{(Avl_{1}+10)\cdot 20\cdot q}, NewcycleT, cycleT_{c}\right]_{2}^{+}, \ 1 \leq c \leq ciclo.$

Step 26

A weight is associated to each animal, at the same time that the objects $XE_{c,i,m,d}$ are generated that keep all the necessary information to start the final phase, fattening.

$r_{106} \equiv \ \left[ZT_{i,m,d}, btt_{c}\right]_{2}^{+} \longrightarrow XE_{c,i,m,d}, WT_{c,i,m,d}^{Tweight_{1+i+j}-m\cdot lossdiseadeT}[\quad]_{2}^{0}, 1 \leq i \leq Tpen, 0 \leq m \leq 1{,}0 \leq d \leq IncT + InfT, 1 \leq c \leq ciclo, 1 \leq j \leq 5.$

$r_{107} \equiv \ [NewcycleT, cycleT_{c}]_{2}^{+} \longrightarrow cycleT'_{c+1}, NewcycleT[\quad]_{2}^{0}, 1 \leq c \leq ciclo.$

$r_{108} \equiv \ [NewcycleT, cycleT_{ciclo}]_{2}^{+} \longrightarrow FinT, NewcycleT[\quad]_{2}^{0}.$

Step 27

Preparation of the objects to start the final phase.

$r_{109} \equiv \ \left[cycleT'_{c} \longrightarrow cycleT_{c}\right]_{0}^{0}, 2 \leq c \leq ciclo.$

$r_{110} \equiv \ [FinT \longrightarrow cycleE_{1}]_{0}^{0}.$

$r_{111} \equiv \ NewcycleT[\quad]_{4}^{+} \longrightarrow [\quad]_{4}^{-}.$

$r_{112} \equiv \ [MT_{i}]_{4}^{-} \longrightarrow MT_{i}[\quad]_{4}^{0}, 1 \leq i \leq q.$

$r_{113} \equiv \ bt_i[\quad]_4^- \longrightarrow [\#]_4^0, 1 \leq i \leq ciclo.$

$r_{114} \equiv \ [btt_i \longrightarrow \#]_2^0, 1 \leq i \leq ciclo.$

$r_{115} \equiv \ [PN'_i]_4^- \xrightarrow{gt_2} PN_i[\quad]_4^0, 1 \leq i \leq floor\left(\frac{Tpen}{2}\right).$

Phase 4: Fattening phase

The model in this last phase is similar to that of the previous phase, transition. The transition phase was carried out in the membrane 2, the fattening phase in the membrane3. It starts with the object $cycleE_c$, which is generated at the end of the previous phase.

Step 1

The first step is to generate objects that will allow the selection of the objects related to the pigs that must evolve and a counter to synchronize the model in this stage.

$r_1 \equiv \; cycleE_c[\quad]_3^0 \longrightarrow be_c^{(Avl_1+4)\cdot q}, RG_0[cycleE_c]_3^-, 1 \leq c \leq ciclo.$

Step 2

The object $WT_{c,i,m,d}$ that was used to record the weight and health status of each pig in the transition phase is eliminated.

$r_2 \equiv \; WT_{c,i,m,d}[\quad]_3^- \longrightarrow [\quad]_3^-, 1 \leq c \leq ciclo, 1 \leq i \leq Tpen, 0 \leq m \leq 2, 0 \leq d \leq IncT + infT.$

The management can be done in two ways: grouping the animals according to the pen of origin or distribute them randomly.

Case 1: Distribution by pens

Step 2

It is accepted that the number of pens in the phase of fattening is double that in the transition phase, so that the pigs of a pen i are divided in the phase of fattening between the pens $2i$ and $2i-1$.

$r_3 \equiv \; be_c, XE_{c,i,1,t}[\quad]_3^- \xrightarrow{ge_1 0.5} \left[IE_{2\cdot i-1,t}\right]_3^0, 1 \leq c \leq ciclo, 1 \leq i \leq Tpen, 1 \leq t \leq IncE + infE.$

$r_4 \equiv \; be_c, XE_{c,i,1,t}[\quad]_3^- \xrightarrow{ge_1 0.5} \left[IE_{2\cdot i,t}\right]_3^0, 1 \leq c \leq ciclo, 1 \leq i \leq Tpen, 1 \leq t \leq IncE + infE.$

$$r_5 \equiv \ be_c, XE_{c,i,1,0}[\quad]_3^- \xrightarrow{ge_1 0.5} [RE_{2\cdot i-1}]_3^0, 1 \leq c \leq ciclo, 1 \leq i \leq Tpen.$$

$$r_6 \equiv \ be_c, XE_{c,i,1,0}[\quad]_3^- \xrightarrow{ge_1 0.5} [RE_{2\cdot i}]_3^0, 1 \leq c \leq ciclo, 1 \leq i \leq Tpen.$$

$$r_7 \equiv \ be_c, XE_{c,i,0,0}[\quad]_3^- \xrightarrow{ge_1 0.5} [SE_{2\cdot i-1}]_3^0, 1 \leq c \leq ciclo, 1 \leq i \leq Tpen.$$

$$r_8 \equiv \ be_c, XE_{c,i,0,0}[\quad]_3^- \xrightarrow{ge_1 0.5} [SE_{2\cdot i}]_3^0, 1 \leq c \leq ciclo, 1 \leq i \leq Tpen.$$

Case 2: Random distribution

Step 2

In this case, objects associated with the pens are generated. These objects will later be associated with objects related to animals.

$$r_9 \equiv \ PNE_i[\quad]_3^- \xrightarrow{ge_2} \left[PNE'_i, PNE_i^{\frac{q\cdot Avl_1-4}{Epen}}\right]_3^-, 1 \leq i \leq Epen.$$

The objects associated with the animals enter the membrane 3, where they will perform the fattening process.

$$r_{10} \equiv \ be_c, XE_{c,p,1,t}[\quad]_3^- \xrightarrow{ge_2} \left[XE'_{1,t}\right]_3^-, 1 \leq c \leq ciclo, 1 \leq p \leq Epen, 0 \leq t \leq IncE + infE.$$

$$r_{11} \equiv \ be_c, XE_{c,p,0,0}[\quad]_3^- \xrightarrow{ge_2} \left[XE'_{0,0}\right]_3^-, 1 \leq c \leq ciclo, 1 \leq p \leq Epen.$$

Step 3 to 9

The animals are distributed by pens.

$$r_{12} \equiv \left[PNE_i, XE'_{1,t} \xrightarrow{ge_2} PNE_i, IE_{i,t}\right]_3^-, 1 \leq i \leq Epen, 1 \leq t \leq IncE + infE.$$

$$r_{13} \equiv \left[PNE_i, XE'_{1,0} \xrightarrow{ge_2} PNE_i, RE_{i,}\right]_3^-, 1 \leq i \leq Epen.$$

$$r_{14} \equiv \left[PNE_i, XE'_{0,0} \xrightarrow{ge_2} PNE_i, SE_{i,}\right]_3^-, 1 \leq i \leq Epen.$$

Counter equal in both cases (Step 2to9)$r_{15} \equiv$

$[RG_i \longrightarrow RG_{i+1}]_0^0,\ 0 \leq i \leq 7.$

$r_{16} \equiv\ RG_8[\quad]_3^- \rightarrow RG_9[\quad]_3^0.$

Common part

<u>*Step 10*</u>

In the case of random distribution, objects PNE_i have been generated that have already carried out their mission, therefore we eliminate them.

$r_{17} \equiv\ [PNE_i]_3^0 \xrightarrow{ge_2} [\#]_3^-, 1 \leq i \leq Epen.$

Evolution of the objects associated with the animals that keep the sanitary state. Infected animals can be found in the incubation phase or in the transmission phase of the disease. The animals at the end of the disease can recover or die. The unit of time with which the model works in this phase is 2 days.

$r_{18} \equiv\ \left[IE_{i,t}\right]_3^0 \longrightarrow \left[IE_{i,t+2}\right]_3^-, 1 \leq i \leq Epen, 0 \leq t \leq IncE.$

$r_{19} \equiv\ \left[IE_{i,t}\right]_3^0 \longrightarrow \left[IE_{i,t+2}, INFGE_i^{KE\cdot ceg}\right]_3^-, 1 \leq i \leq Epen, IncE < t \leq IncE + infE -$
2.

$r_{20} \equiv\ \left[IE_{i,t}\right]_3^0 \xrightarrow{(1-LE)} [RE_i]_3^-, 1 \leq i \leq Epen, IncE + infE - 2 < t \leq IncE + infE.$

$r_{21} \equiv\ \left[IE_{i,t}\right]_3^0 \xrightarrow{LE} [LE_i]_3^-, 1 \leq i \leq Epen, IncE + infE - 2 < t \leq IncE + infE.$

Evolution counter

$r_{22} \equiv\ RG_9[\quad]_3^0 \longrightarrow RG_{10}[\quad]_3^-.$

<u>*Step 11*</u>

Infectious elements evolve and are distributed within the farm, the contagion risk is greater than short distances from the contagion site.

$r_{23} \equiv\ \left[INFGE_i \xrightarrow{\frac{2}{7}} INFE_i\right]_3^-, 1 < i < Epen, i \neq \frac{Epen}{2}, i \neq \frac{Epen}{2} + 1.$

$$r_{24} \equiv \left[INFGE_i \xrightarrow{\frac{1}{7}} INFE_{i-1}\right]_3^{-}, 1 < i < Epen, i \neq \frac{Epen}{2}, i \neq \frac{Epen}{2} + 1.$$

$$r_{25} \equiv \left[INFGE_i \xrightarrow{\frac{1}{7}} INFE_{i+1}\right]_3^{-}, 1 < i < Epen, i \neq \frac{Epen}{2}, i \neq \frac{Epen}{2} + 1.$$

$$r_{26} \equiv \left[INFGE_i \xrightarrow{\frac{1}{7}} INFE_{Epen-i+1}\right]_3^{-}, 1 < i < Epen, i \neq \frac{Epen}{2}, i \neq \frac{Epen}{2} + 1.$$

$$r_{27} \equiv \left[INFGE_i \xrightarrow{\frac{1}{7}} INFE_{Epen-i+2}\right]_3^{-}, 1 < i < Epen, i \neq \frac{Epen}{2}, i \neq \frac{Epen}{2} + 1.$$

$$r_{28} \equiv \left[INFGE_i \xrightarrow{\frac{1}{7}} INFE_{Epen-i}\right]_3^{-}, 1 < i < Epen, i \neq \frac{Epen}{2}, i \neq \frac{Epen}{2} + 1.$$

$$r_{29} \equiv \left[INFGE_1 \xrightarrow{\frac{2}{5}} INFE_1\right]_3^{-}.$$

$$r_{30} \equiv \left[INFGE_1 \xrightarrow{\frac{1}{5}} INFE_2\right]_3^{-}.$$

$$r_{31} \equiv \left[INFGE_1 \xrightarrow{\frac{1}{5}} INFE_{Epen}\right]_3^{-}.$$

$$r_{32} \equiv \left[INFGE_1 \xrightarrow{\frac{1}{5}} INFE_{Epen-1}\right]_3^{-}.$$

$$r_{33} \equiv \left[INFGE_{\frac{Epen}{2}} \xrightarrow{\frac{2}{5}} INFE_{\frac{Epen}{2}}\right]_3^{-}.$$

$$r_{34} \equiv \left[INFGE_{\frac{Epen}{2}} \xrightarrow{\frac{1}{5}} INFE_{\frac{Epen}{2}-1}\right]_3^{-}.$$

$$r_{35} \equiv \left[INFGE_{\frac{Epen}{2}} \xrightarrow{\frac{1}{5}} INFE_{\frac{Epen}{2}+1}\right]_3^{-}.$$

$$r_{36} \equiv \left[INFGE_{\frac{Epen}{2}} \xrightarrow{\frac{1}{5}} INFE_{\frac{Epen}{2}+2}\right]_3^{-}.$$

$$r_{37} \equiv \left[INFGE_{\frac{Epen}{2}+1} \xrightarrow{\frac{2}{5}} INFE_{\frac{Epen}{2}+1}\right]_3^{-}.$$

$$r_{38} \equiv \left[INFGE_{\frac{Epen}{2}+1} \xrightarrow{\frac{1}{5}} INFE_{\frac{Epen}{2}-1}\right]_3^{-}.$$

$$r_{39} \equiv \left[INFGE_{\frac{Epen}{2}+1} \xrightarrow{\frac{1}{5}} INFE_{\frac{Epen}{2}}\right]_3^{-}.$$

$$r_{40} \equiv \left[INFGE_{\frac{Epen}{2}+1} \xrightarrow{\frac{1}{5}} INFE_{\frac{Epen}{2}+2}\right]_3^{-}.$$

$$r_{41} \equiv \left[INFGE_{Epen} \xrightarrow{\frac{2}{5}} INFE_{Epen}\right]_3^{-}.$$

$$r_{42} \equiv \left[INFGE_{Epen} \xrightarrow{\frac{1}{5}} INFE_{1}\right]_3^{-}.$$

$$r_{43} \equiv \left[INFGE_{Epen} \xrightarrow{\frac{1}{5}} INFE_{2}\right]_3^{-}.$$

$$r_{44} \equiv \left[INFGE_{Epen} \xrightarrow{\frac{1}{5}} INFE_{Epen-1}\right]_3^{-}.$$

Evolution of the counter that generates $daye$ objects that records the number of days since the beginning of the fattening phase, and therefore indirectly the age of the animal.

$$r_{45} \equiv [RG_{10} \longrightarrow RG_{11}, daye]_0^0.$$

<u>*Step 12*</u>

Phase of transmission of the disease.

$$r_{46} \equiv IE_{i,t}, INFE_i[\quad]_{33}^0 \longrightarrow \left[IE_{i,t}\right]_{33}^{+}, 1 \le i \le Epen, 0 \le t \le IncE + InfE.$$

$$r_{47} \equiv RE_{i,t}, INFE_i[\quad]_{33}^0 \longrightarrow [RE_i]_{33}^{+}, 1 \le i \le Epen.$$

$$r_{48} \equiv SE_{i,t}, INFE_i[\quad]_{33}^0 \xrightarrow{(pE\cdot 2-pE\cdot pE)} \left[IE_{i,0}\right]_{33}^{+}, 1 \le i \le Epen.$$

$$r_{49} \equiv SE_{i,t}, INFE_i[\quad]_{33}^0 \xrightarrow{(1-pE\cdot 2-pE\cdot pE)} [SE_i]_{33}^{+}, 1 \le i \le Epen.$$

When 60 objects of the $daye$ type have accumulated, 120 days have passed and it is the moment when the animal finishes the fattening phase.

$$r_{50} \equiv [daye^{60} \longrightarrow ende]_0^0.$$

$r_{51} \equiv [RG_{11} \longrightarrow RG_{12}]_0^0.$

Step 13

The transmission of the disease has been made in the membrane labeled by 33, to follow the process these objects must leave this membrane.

$r_{52} \equiv [IE_{i,t}]_{33}^{+} \longrightarrow IE_{i,t}[\quad]_{33}^{0}, 1 \leq i \leq Epen, 0 \leq t \leq IncE + infE + 1.$

$r_{53} \equiv [RE_i]_{33}^{+} \longrightarrow RE_i[\quad]_{33}^{0}, 1 \leq i \leq Epen.$

$r_{54} \equiv [SE_i]_{33}^{+} \longrightarrow SE_i[\quad]_{33}^{0}, 1 \leq i \leq Epen.$

$r_{55} \equiv [LE_i]_{33}^{+} \longrightarrow LE_i[\quad]_{33}^{0}, 1 \leq i \leq Epen.$

$r_{56} \equiv INFE_i[\quad]_{33}^{+} \longrightarrow [\#]_{33}^{0}, 1 \leq i \leq Epen.$

The evolution of the counter depends on whether or not the end of the fattening phase has been reached.

$r_{57} \equiv RG_{12}, ende[\quad]_3^{-} \longrightarrow RG_{13}[ende]_3^{+}.$

$r_{58} \equiv RG_{12}, daye[\quad]_3^{-} \longrightarrow RG_9, daye[\quad]_3^{0}.$

Step 14

In the case of reaching the end of the fattening phase for pigs of batch , the system configuration must be restored in order to start the process for the animals of the following batch.

$r_{59} \equiv [PNE'_i]_3^{+} \xrightarrow{ge_2} PNE_i[\#]_3^{+}, 1 \leq i \leq Epen.$

$r_{60} \equiv RG_{13}[\quad]_3^{+} \longrightarrow [RG_{14}]_3^{+}.$

$r_{61} \equiv ende[\quad]_{33}^{0} \longrightarrow [\#]_{33}^{+}.$

Step 15

Evolution of the objects that register the health status to objects $ZE_{i,j}$, the first index keeps the pen in which the animal is found and the second the health status.

$r_{62} \equiv\ SE_i[\quad]_{33}^{+} \longrightarrow \left[ZE_{i,0}\right]_{33}^{0}, 1 \leq i \leq Epen.$

$r_{63} \equiv\ IE_{i,t}[\quad]_{33}^{+} \longrightarrow \left[ZE_{i,1}\right]_{33}^{0}, 1 \leq i \leq Epen, 0 \leq t \leq IncE.$

$r_{64} \equiv\ IE_{i,t}[\quad]_{33}^{+} \longrightarrow \left[ZE_{i,2}\right]_{33}^{0}, 1 \leq i \leq Epen, IncE + 1 \leq t \leq IncE + InfE.$

$r_{65} \equiv\ RE_i[\quad]_{33}^{+} \longrightarrow \left[ZE_{i,3}\right]_{33}^{0}, 1 \leq i \leq Epen.$

$r_{66} \equiv\ LE_i[\quad]_{33}^{+} \longrightarrow [\#]_{33}^{0}, 1 \leq i \leq Epen.$

Evolution of the counter.

$r_{67} \equiv\ [RG_{14} \longrightarrow RG_{15}]_{3}^{+}$

Step 16

Natural mortality

$r_{68} \equiv\ \left[ZE_{i,0}\right]_{33}^{0} \xrightarrow{1-me} ZE_{i,0}[\quad]_{33}^{0}, 1 \leq i \leq Epen.$

$r_{69} \equiv\ \left[ZE_{i,1}\right]_{33}^{0} \xrightarrow{1-me} ZE_{i,1}[\quad]_{33}^{0}, 1 \leq i \leq Epen.$

$r_{70} \equiv\ \left[ZE_{i,2}\right]_{33}^{0} \xrightarrow{1-me} ZE_{i,2}[\quad]_{33}^{0}, 1 \leq i \leq Epen.$

$r_{71} \equiv\ \left[ZE_{i,3}\right]_{33}^{0} \xrightarrow{1-me} ZE_{i,3}[\quad]_{33}^{0}, 1 \leq i \leq Epen.$

$r_{72} \equiv\ \left[ZE_{i,0}\right]_{33}^{0} \xrightarrow{me} [\#]_{33}^{0}, 1 \leq i \leq Epen.$

$r_{73} \equiv\ \left[ZE_{i,1}\right]_{33}^{0} \xrightarrow{me} [\#]_{33}^{0}, 1 \leq i \leq Epen.$

$r_{74} \equiv\ \left[ZE_{i,2}\right]_{33}^{0} \xrightarrow{me} [\#]_{33}^{0}, 1 \leq i \leq Epen.$

$r_{75} \equiv\ \left[ZE_{i,3}\right]_{33}^{0} \xrightarrow{me} [\#]_{33}^{0}, 1 \leq i \leq Epen.$

Evolution of the counter, if the end of the cycle has been reached, objects will be generated that will allow the next cycle to start.

$r_{76} \equiv\ \left[RG_{15}, cycleE_c \rightarrow bee_c^{(Avl_1+2)\cdot q}, NewcycleE, cycleE_c\right]_{3}^{+}, 1 \leq c \leq ciclo.$

Step 17

The objects that keep the information of the sanitary status of the animal and the weight reached at the end of the fattening phase are generated.

$r_{77} \equiv \left[ZE_{i,d}, bee_c\right]_3^+ \rightarrow ZF_{c,i,d}, WE_{c,i,d}^{Eweight(1+i\cdot j)}[\quad]_3^0, 1 \leq i \leq Epen, 0 \leq d \leq 3, d \neq 2, \neq 1 \leq c \leq ciclo, 1 \leq j \leq 5.$

$r_{78} \equiv \left[ZE_{i,2}, bee_c\right]_3^+ \rightarrow ZF_{c,i,2}, WE_{c,i,2}^{Eweight(1+i\cdot j)-lossdiseadeE}[\quad]_3^0, 1 \leq i \leq Epen, 1 \leq c \leq ciclo, 1 \leq j \leq 5.$

Evolution of objects related to the simulated cycle.

$r_{79} \equiv [NewcycleE, cycleE_c]_3^+ \longrightarrow cycleE'_{c+1}, NewcycleE[\quad]_3^0, 1 \leq c \leq ciclo.$

$r_{80} \equiv [NewcycleE, cycleE_{ciclo}]_3^+ \longrightarrow NewcycleE[\quad]_3^0.$

Step 18

Elimination of leftover objects and preparation to start the next cycle until reaching the end of the model.

$r_{81} \equiv [cycleE'_c \longrightarrow cycleE_c]_0^0, 2 \leq c \leq ciclo.$

$r_{82} \equiv NewcycleE\ [\quad]_4^0 \longrightarrow [\#]_4^-.$

$r_{83} \equiv be_i\ [\quad]_4^- \longrightarrow [\#]_4^-, 1 \leq c \leq ciclo.$

$r_{84} \equiv [bee_i \longrightarrow \#]_3^0, 1 \leq c \leq ciclo.$
